# Systematic analysis of cell morphodynamics in *C. elegans* early embryogenesis

**DOI:** 10.3389/fbinf.2023.1082531

**Published:** 2023-03-21

**Authors:** Yusuke Azuma, Hatsumi Okada, Shuichi Onami

**Affiliations:** Laboratory for Developmental Dynamics, RIKEN Center for Biosystems Dynamics Research, Kobe, Japan

**Keywords:** morphodynamics, bioimage informatics, *C. elegans*, quantitative biology, reproducibility, cell-cell contact, mitotic rounding

## Abstract

The invariant cell lineage of *Caenorhabditis elegans* allows unambiguous assignment of the identity for each cell, which offers a unique opportunity to study developmental dynamics such as the timing of cell division, dynamics of gene expression, and cell fate decisions at single-cell resolution. However, little is known about cell morphodynamics, including the extent to which they are variable between individuals, mainly due to the lack of sufficient amount and quality of quantified data. In this study, we systematically quantified the cell morphodynamics in 52 *C. elegans* embryos from the two-cell stage to mid-gastrulation at the high spatiotemporal resolution, 0.5 μm thickness of optical sections, and 30-second intervals of recordings. Our data allowed systematic analyses of the morphological features. We analyzed sphericity dynamics and found a significant increase at the end of metaphase in every cell, indicating the universality of the mitotic cell rounding. Concomitant with the rounding, the volume also increased in most but not all cells, suggesting less universality of the mitotic swelling. Combining all features showed that cell morphodynamics was unique for each cell type. The cells before the onset of gastrulation could be distinguished from all the other cell types. Quantification of reproducibility in cell-cell contact revealed that variability in division timings and cell arrangements produced variability in contacts between the embryos. However, the area of such contacts occupied less than 5% of the total area, suggesting the high reproducibility of spatial occupancies and adjacency relationships of the cells. By comparing the morphodynamics of identical cells between the embryos, we observed diversity in the variability between cells and found it was determined by multiple factors, including cell lineage, cell generation, and cell-cell contact. We compared the variabilities of cell morphodynamics and cell-cell contacts with those in ascidian *Phallusia mammillata* embryos. The variabilities were larger in *C. elegans*, despite smaller differences in embryo size and number of cells at each developmental stage.

## 1 Introduction

*Caenorhabditis elegans* is one of the best-characterized model organisms to study animal development. Its development proceeds through an invariant cell lineage, namely, the stereotypical pattern of cell divisions, and produces an adult hermaphrodite with just 959 somatic cells. The whole-cell lineage was first established by Sulston *et al.* by manually tracking cells with differential interference contrast (DIC) microscopy (Sulston et al., 1983). Recent advances in bioimage informatics enabled automated tracing of the cell lineage (Onami et al., 2001; Bao et al., 2006). Typical studies perform 4D (3D time-lapse) imaging of embryos with fluorescently labeled nuclei (typically histone) and computationally identify and track the nuclei (Azuma and Onami, 2013). In addition to the nuclear labeling, the use of reporter genes enables the measurement of the reporter expression dynamics at single-cell resolution. This method provides reporter dynamics with lineage information. It allowed systematic analysis of developmental dynamics, including variability of cell division timings and cell cycle lengths measured in 20 embryos (Bao et al., 2008), reproducibility of cell cycle lengths, division axes, and cell positions measured in 18 embryos (Richards et al., 2013), gene expression dynamics in 127 cells (Murray et al., 2012), high-dimensional phenotypic analysis of 204 essential genes in 1,368 perturbed embryos (Du et al., 2015), and lineage-specificity of variability in cell positions (Li et al., 2019).

Cell morphology is also associated with a variety of biological processes. Relationships have been found between cell volume and cell cycle length (Arata et al., 2015), cell volume and strength of the spindle assembly checkpoint (Galli and Morgan, 2016), asymmetric divisions and the local inactivation of actomyosin cortical contractility (Rose and Gönczy, 2013), and asymmetric divisions and confinement of embryos (Fickentscher and Weiss, 2017). In addition to nuclear labeling, membrane reporters enable systematic analysis of cell morphodynamics. However, there have been few such studies due to the difficulty in cell membrane segmentation, which is more challenging than nuclei. This is because nuclei are thick, well-separated spherical structures, whereas cell membranes are thin planar structures that contact each other, forming complicated networks. Despite these difficulties, we succeeded in developing the membrane segmentation method called BCOMS (Biologically Constrained Optimization-based cell Membrane Segmentation). It automatically segments cell membranes and extracts morphological features of each cell by solving an objective function under biological constraints (Azuma and Onami, 2017). It uses previously detected nuclei as markers after manual curation, yielding cell segmentations with no missed cells. The performance of BCOMS was evaluated by comparisons with manually created ground truth and between two adjacent time points and was the best among the available methods. In addition, recent advances in deep learning brought image restoration to a practical level (Weigert et al., 2018). In combination with this technique, we may improve the quantification accuracy because it is dependent on the membrane image quality.

In this study, we developed an image processing pipeline to quantify cell morphodynamics by combining previously and newly developed computational methods, including nuclear detection and tracking, image restoration, and membrane segmentation. We applied the pipeline for 52 *C. elegans* embryos and systematically extracted morphological features. At first, we show that we can understand developmental dynamics quantitatively by systematic analysis of the extracted features. Next, we show that our data can reproduce and extend a previous study. Finally, we obtain biological insights about cell morphodynamics by comparisons with the studies of *C. elegans* and ascidian *Phallusia mammillata* embryos.

## 2 Results

### 2.1 Image processing

We investigated whether existing methods are available for this study. At first, we evaluated whether an existing image restoration method was effective for our images. There is an image restoration method called CARE (Weigert et al., 2018). To apply CARE, we need to prepare training data. We prepared registered pairs of low- and high-quality images acquired by quickly changing laser power and exposure time. The images were acquired in sparser spatial and temporal resolutions than actual settings to prevent photobleaching and for unbiased sampling throughout development and across optical sections (Supplementary Figure S1). We acquired the images of 10 embryos. CARE was trained using a part (90%) of the prepared data and applied to the test data, the remaining prepared data. We found artifacts were introduced in some images (Supplementary Figure S2). Especially more artifacts were observed in deeper optical sections, where images are more degraded than in shallow sections by light scattering and absorption. To solve this problem, we developed a model named restworm by modifying the U-Net (Ronneberger et al., 2015) used in CARE (see Methods). Restworm was trained on the same training data and applied to the test data. We found that the artifacts were removed (Supplementary Figure S2). We also confirmed that membrane segmentation using the restored images did not derive any artifacts compared to the original membrane images (Supplementary Figure S3).

Next, we evaluated whether existing membrane segmentation methods are available for our study. There are three such methods developed after the BCOMS, namely, 3DMMS (Cao et al., 2019), CShaper (Cao et al., 2020), and spheresDT/Mpacts-PiCS (Thiels et al., 2021). We found that they all perform membrane segmentation solely from membrane images and have no nucleus information. Additional software is needed to obtain cell lineage information. Moreover, these methods cannot control false detections. Thus, some cells may be lost. In contrast, BCOMS performs membrane segmentation using detected nuclei as markers. It ensures no cells are missed if the nuclear detection does not miss any nucleus. In the BCOMS pipeline, we created errorless nucleus detections by curating output from the previously developed software (Azuma and Onami, 2013) using in-house curation software named Curater. Curater also has a function to identify cell lineage and annotate the nuclei. The function uses publicly available annotated data (Richards et al., 2013) as a reference and annotates all cells detected (see Methods for details). However, BCOMS had some missing functions. Hence, we decided to use the BCOMS for this study after adding the functions, which we call BCOMS2. One added function is the collection of the time lag between nuclear divisions (karyokinesis) and cell divisions (cytokinesis). In this period, the cell is divided into two regions using the two divided nuclei as markers, whereas the cell is before the division. The added function prevents incorrect segmentation by collecting the time lag by a machine learning-based method (see Methods). The other added function is the extraction of cell-cell contacts. These are detected from the cell membrane segmentation results as pairs of cells contacting their surfaces. For each pair, BCOMS2 extracts the contact area and duration.

**FIGURE 1:**
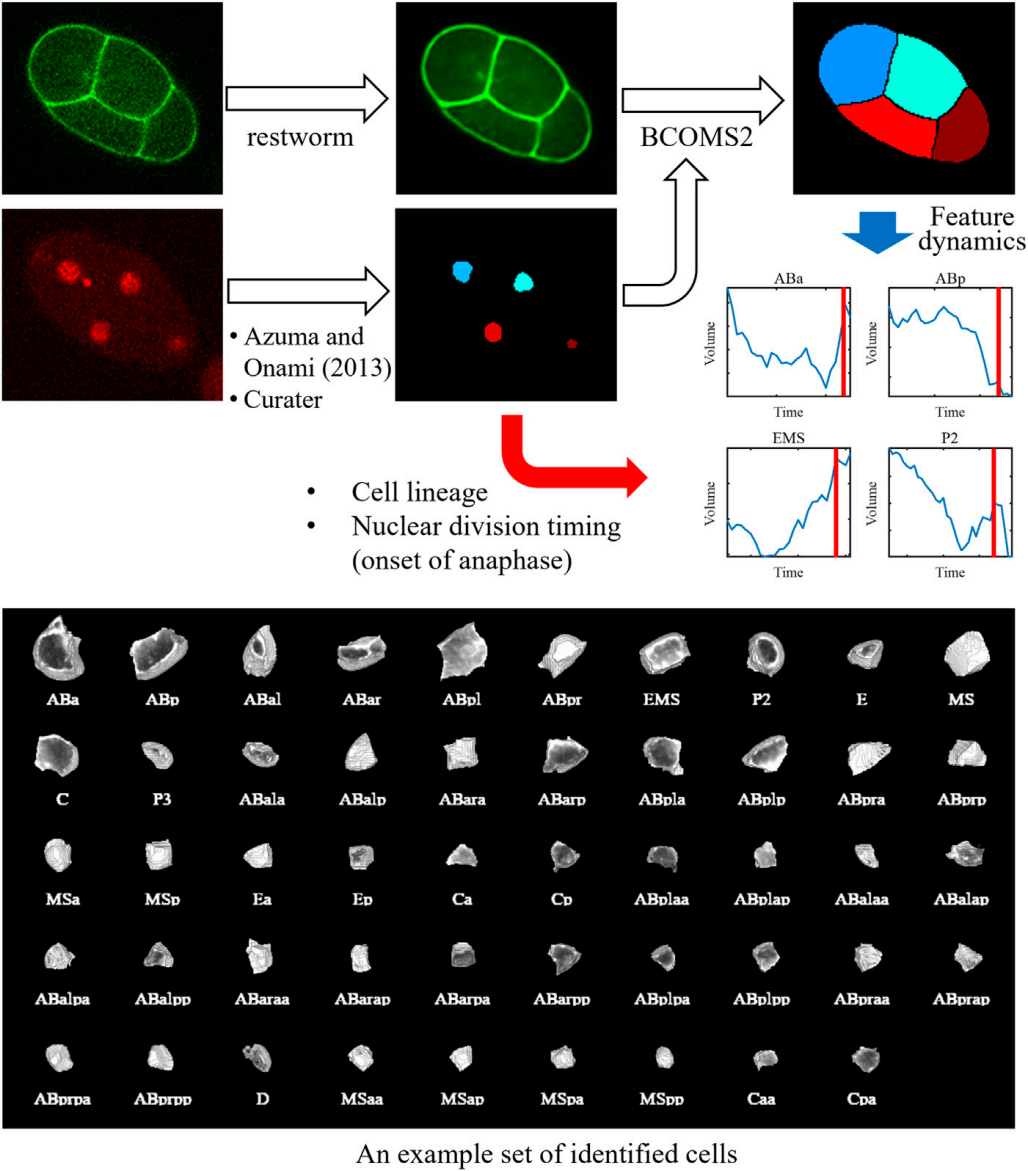
Schematic of the image processing pipeline and an exemplary segmentation result. The nucleus image is processed using a nucleus detection method developed previously. The result is manually curated using Curater, providing errorless nucleus detections. Each nucleus is also annotated using Curater. The membrane image is denoised using restworm. The restored membrane image and processed nucleus detection results are input to BCOMS2. BCOMS2 performs membrane segmentation and extracts morphological features, providing feature dynamics of the identified cells. The annotations for the nuclei are assigned for the cells, and the nuclear division timings are used to provide the timings of anaphase onset.

We combined the methods and developed an image processing pipeline to quantify cell morphodynamics (Figure 1). In this pipeline, the nucleus image is processed using a nucleus detection method (Azuma and Onami, 2013). The result is manually curated using Curater, providing errorless nucleus detections. Each detected nucleus is also annotated using Curater. In parallel, the membrane image is restored by restworm. The restored membrane image and processed nucleus detection results are input to BCOMS2. BCOMS2 performs membrane segmentation using detected nuclei as markers and extracts morphological features from the segmented cells (Supplementary Table S1). Meanwhile, the annotations for the nuclei are assigned for the identified cells, and the nuclear division timings are used to provide the timings of anaphase onset. All the software developed in this study can be available from https://github.com/bioimage-informatics.

We performed 3D timelapse imaging of 52 embryos, in which the nucleus and cell membrane were labeled with green fluorescent protein (GFP) and mCherry, respectively, for 2 hours from the two-cell stage. We applied the image processing pipeline (Figure 1) for the 52 embryos (the results of ABp are shown in Supplementary Figure S4, and a segmentation result in an embryo is shown in Supplementary Video S1) and extracted morphological features (Supplementary Table S1). All images and segmentation results are available via SSBD:repository (Tohsato et al., 2016) (https://doi.org/10.24631/ssbd.repos.2022.06.236). We evaluated the performance by calculating the volume deviation between two adjacent time points used in the previous study (Azuma and Onami, 2017). Comparing with manual segmentation results at multiple time points, we confirmed that systematic error was not introduced (Azuma and Onami, 2017). Thus, it can be used as an indicator to evaluate the accuracy of the segmentation results, especially for this data where the time interval is short (30 s) enough to regard the volume as nearly stable between the adjacent time points. The deviation was reduced by 8% (from 0.065 in BCOMS to 0.060 in BCOMS2), demonstrating improved quantification accuracy.

The developmental rates can vary even if the imaging conditions are consistent (Schnabel et al., 1997; Bao et al., 2008). The final developmental stages differed between the embryos for the same duration of the recordings. Indeed, the ratio between the fastest and slowest rates was 1.2 in our data. As a result, the number of cells at the final time point varied from 51 to 96, and the number of cells that completed their cell cycle varied from 49 to 119 (Supplementary Figure S5). Of the 52 embryos, 32 exceeded the 85-cell stage and contained at least 76 cell types (the same cell names) that completed the cell cycle. We used the data from the 52 or 32 embryos for the following analyses.

### 2.2 Quantitative analysis of cell morphodynamics

**FIGURE 2:**
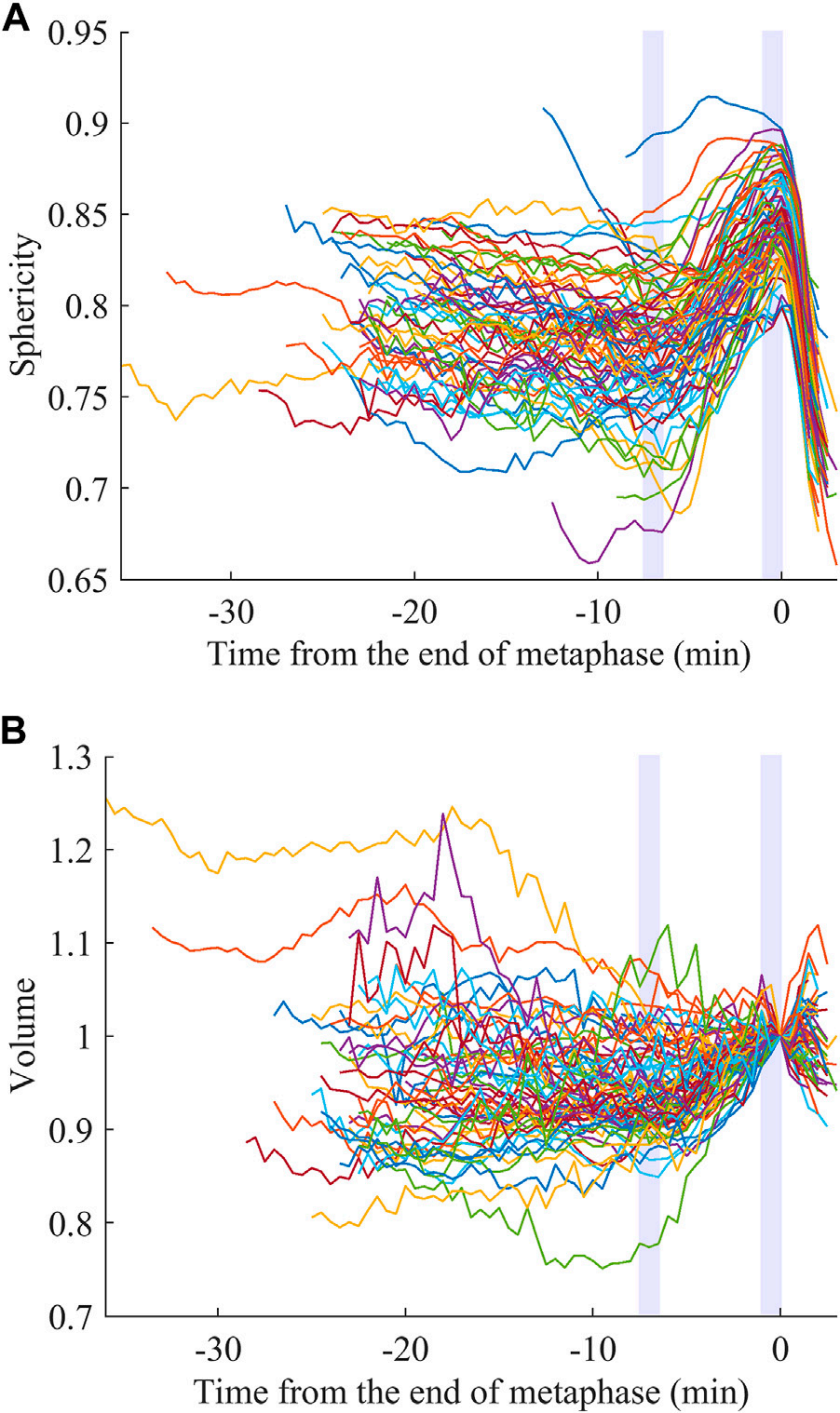
Mitotic rounding and swelling. Sphericity **(A)** and volume **(B)** dynamics averaged for the 32 embryos registered at the end of metaphase. The light purple rectangles indicate the initial and end of the estimated duration of the mitotic rounding.

We examined whether the extracted features are related to specific biological processes and used for obtaining biological insights. We focused on sphericity and volume. It is known that animal cells round up to become spherical when dividing, which is called mitotic cell rounding. It has been commonly observed *in vivo* during development in many animals, including mouse (Luxenburg et al., 2011), fly (Kondo and Hayashi, 2013; Rosa et al., 2015; Chanet et al., 2017), and zebrafish (Hoijman et al., 2015). However, little is known about *C. elegans*, including in which cells the rounding occurs. The rounding begins at prophase and the rounded shape is assumed during metaphase until the onset of anaphase (Ramkumar and Baum, 2016; Taubenberger et al., 2020). Therefore, if the mitotic rounding occurs in the cells of *C. elegans* embryos, the sphericity is expected to be higher, at least at late metaphase. We registered the sphericity dynamics at the end of metaphase and averaged them at each time point over the 32 embryos for the 76 types of cells. We observed rapid increases of the sphericity at late metaphase in most cell types (Figure 2A). The increase was sharp without a plateau and the peak was within 1.0 min before the end of metaphase in most cell types (74/76, Supplementary Figure S6A). Since the time interval of imaging was 0.5 min, the result suggests that the cells kept rounding until the very end of metaphase. The 2 cell types, ABa and ABp, were an exception and reached a plateau soon after beginning the mitotic rounding, around −4.5 min (Supplementary Figure S6B). The peaks were located several minutes earlier than the end of metaphase. We averaged the dynamics over all cell types and found that the sphericity monotonically increased from −7.0 min, when the sphericity is minimum, to 0 min (Supplementary Figure S6C). We defined this period as the duration of the mitotic rounding. We compared sphericity between the beginning (−7.5 to −6.5 min) and the end (−1.0 to 0 min) of this period in each cell. The sphericity significantly increased in all the cell types from 2.0% to 33% (average, 12%) during this period (paired *t*-test, *p* < 0.05). As the increase was slight in some cells, detection by the human eye is nearly impossible, highlighting the ability of the quantified cell morphology resource.

As a similar event during cell division, mitotic swelling is known. It had been controversial whether cells increased or decreased their volume during mitosis. In 2015, two studies on the same issue developed distinct methods to precisely measure the volume dynamics of adherent or suspended cells and observed cell volume increases (Son et al., 2015; Zlotek-Zlotkiewicz et al., 2015). The increase was observed in cells from a variety of tissues in human and mouse (Zlotek-Zlotkiewicz et al., 2015). However, it is unclear whether the mitotic swelling occurs *in vivo*, especially in confined environments, including the *C. elegans* embryo enclosed in an eggshell. We registered the volume dynamics at the end of metaphase like the sphericity (Figure 2B, close-up in Supplementary Figure S7A; normalized by dividing by the volume at t = 0). Interestingly, the volume dynamics averaged for all cell types reached a minimum at −7.0 min (Supplementary Figure S7B), consistent with the sphericity dynamics (Supplementary Figure S6C). During this period, most types of cells (71/76) significantly swelled from 1.9% to 36% (average, 9.6%; paired *t*-test, *p* < 0.05). In contrast to the mitotic rounding observed in all cell types, the mitotic swelling was not observed in some cell types, suggesting less universality.

**FIGURE 3:**
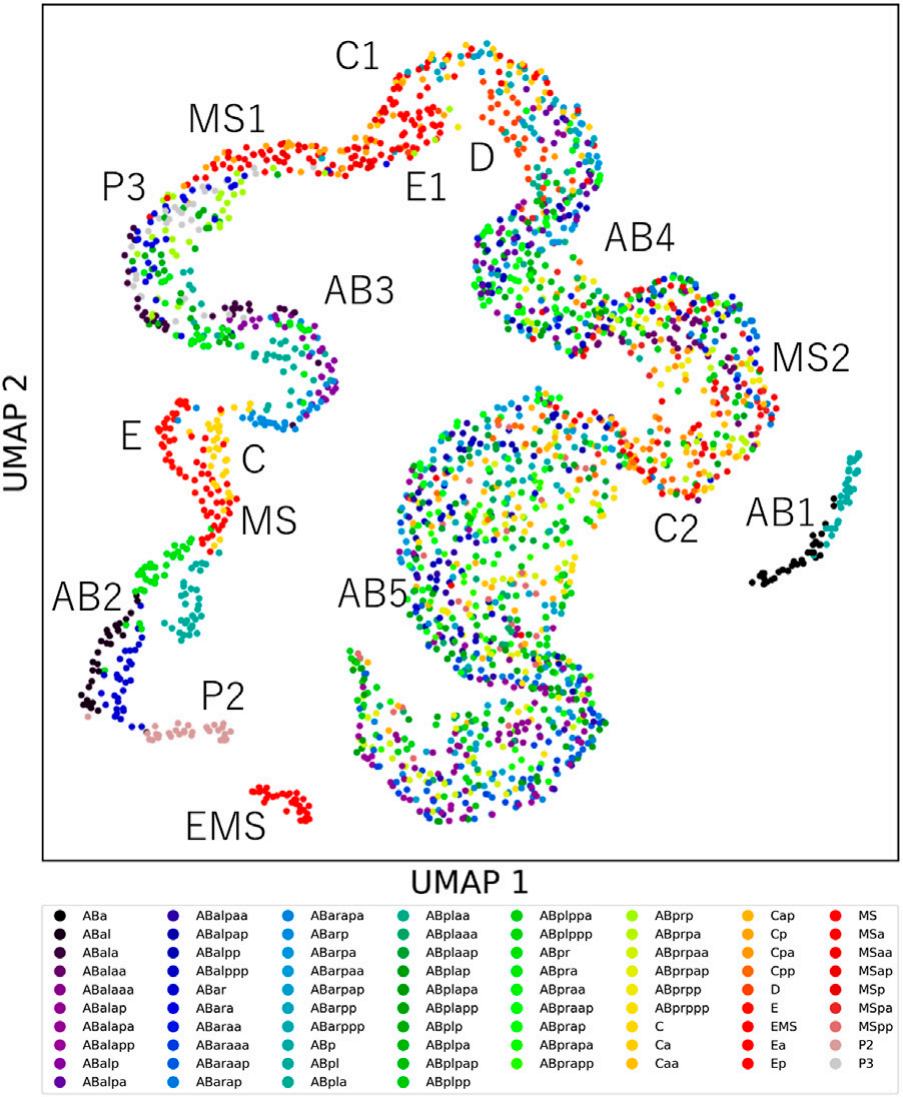
The uniqueness of cell morphodynamics. UMAP projection of the 76 types of cells in the 32 embryos by using the integral RMSDs as the distance matrix. Colors indicate the cell types. Labels indicate the generation names of the cells, represented by lineage names and division times from the founder cell, such as AB2 for ABal, ABar, ABpa, and ABpr.

Cells showing unique feature dynamics raise the possibility that each cell can be distinguished from the other cells by its morphodynamics. If morphodynamics is significantly similar in the same cell types than in different cell types, the cell types can be distinguished from the other cell types. To test this hypothesis, we measured the root mean squared error (RMSE) between feature dynamics of every pair of cells in the 32 embryos after normalizing cell cycle lengths (Methods). We normalized each RMSE by dividing it by the average of the feature dynamics and summed it across all features. We compared the RMSEs and found that 97% of cell types could be distinguished from 95% of the other cell types (Supplementary Figure S8A). We visualized the similarity relationships with the uniform manifold approximation and projection (UMAP) (McInnes et al., 2018; Becht et al., 2019) by using the RMSEs as the distance matrix (Figure 3). We observed approximately one continuous trajectory where the cells were in order of birth timing (Supplementary Figure S8B). The different cell types were separated well in earlier generations and were increasingly mixed along with the progression of embryogenesis. We found that all cell types before the onset of gastrulation were distinguishable from any other cell types except for P4. P4 was not included in the analysis because it did not complete the cell cycle in the 32 embryos.

Following the analysis of single-cell features, we analyzed inter-cell features related to cell-cell contact. In *C. elegans*, cell-cell interactions play essential roles in embryogenesis. Notch signaling is such an interaction and plays a significant role in specifying cell fates and tissue morphogenesis (Priess, 2005). It requires cell-cell contacts to transmit the signal (Kopan and Ilagan, 2009). Similarly, Wnt signaling requires cell-cell contacts for signal transmission (Walston et al., 2004). We examined whether cell-cell contacts mediating the cell-cell interactions were reproducible in all embryos. We quantified the reproducibility of cell-cell contact as the number of embryos where the contact is formed divided by the total number of embryos. As five rounds of Notch signaling and two of Wnt signaling are known during our data, we computed the reproducibility of these contacts (Supplementary Table S2). As expected, the reproducibilities of these contacts were 100%, meaning they were formed in all embryos. Note that the reproducibility of 4th and 5th Notch signaling was measured in a part of the embryos because the cell cycle was completed in only those embryos (Supplementary Table S2).

**FIGURE 4:**
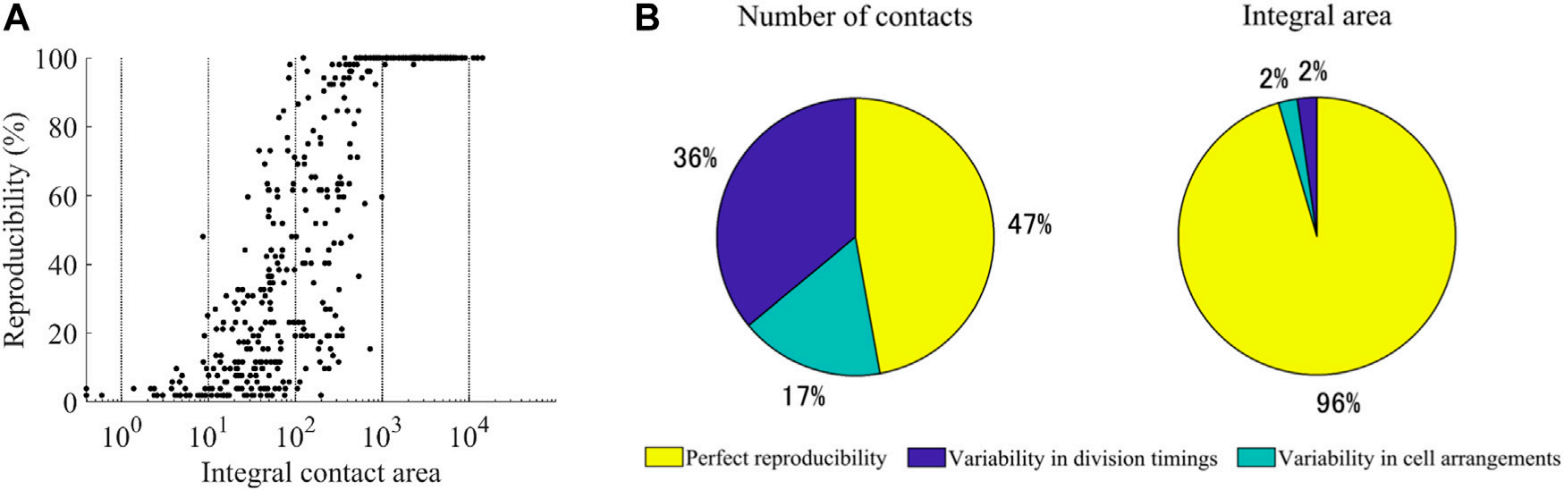
Reproducibility of cell-cell contacts **(A)** Relationship between the reproducibility and the integral area of the contacts. The integral area is shown on a logarithmic scale **(B)** Estimated proportions of the three categories of contacts. The number of contacts (left) and integral area (right) in each category are given as a percentage of the total number and total area of all detected contacts, respectively.

This analysis raised the question of to what extent the contacts were reproducible between the embryos. We measured the reproducibility for the 47 types of cells completing the cell cycle in the 52 embryos. We also measured the integral area by summing the contact area across the cell cycle. We found a biphasic relationship between the integral area and reproducibility (Figure 4A). Above 1,000 μm^2^, most contacts were perfectly reproducible. Only two contacts (ABplap and ABalpp; ABplap and ABplpp) were variable (imperfectly reproducible). We manually checked the images of these contacts and found that they were lost in an embryo throughout their cell cycle due to differences in cell arrangements (Supplementary Videos S2, S3). Below 1,000 μm^2^, the relationship was correlative (r = 0.61), and most (92%) of the contacts were variable. One expected source of the variability is the variability in division timing. During *C. elegans* embryogenesis, the division timing is approximately synchronized in cells of each lineage (Sulston et al., 1983) and slightly varies between embryos (Richards et al., 2013). This variability can generate variability in contacts. We detected such contacts by virtually shifting the division timings back and forth. As a result, we found that 25% of the variable contacts were caused by the variability in division timing. The remaining variable contacts should be caused by false detections or variability in cell arrangements. We randomly selected 10% (23 from 230) of the contacts and manually checked them (Supplementary Table S3). More than half (56.5%, 13/23) were caused by variability in cell arrangements, and false detections caused the others. Based on this result, we estimated the proportions of three categories of contacts: variable contacts caused by variability in contact timing, variable contacts caused by variability in cell arrangement, and perfectly reproducible contacts (Figure 4B). The number of perfectly reproducible contacts was not more than half of all contacts. In contrast, the integral area of such contacts accounted for over 95% of the total area. In contrast, the number of variable contacts were more than half, whereas the corresponding integral area was less than 5%. These results suggest that the spatial occupancy of each cell is highly reproducible, while slight variability in division timings and cell arrangements produces brief variable contacts.

### 2.3 Reproduction and extension of a previous study

**FIGURE 5:**
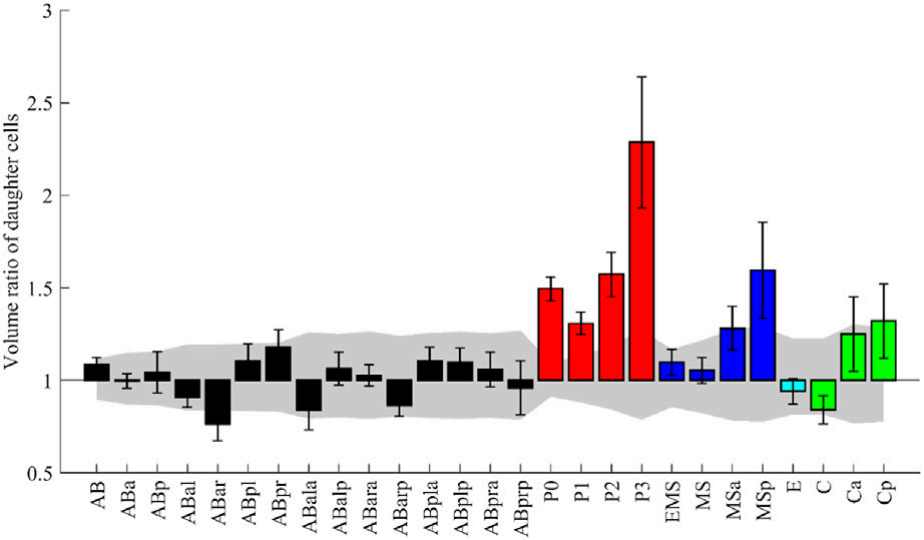
The volume ratio of daughter cells emerging from the named mother cell. The design of the graph follows (Fickentscher and Weiss, 2017). The medians of the 52 embryos are shown with error bars indicating standard deviations. Colors indicate cell lineages. The gray region indicates the volume-dependent level of uncertainty (see Methods for details).

Some morphological features have already been analyzed in *C. elegans* embryos. We picked up one such study and examined whether our data was consistent with their results and could extend the study. The study measured volume asymmetry between daughter cells in all cell divisions until the onset of gastrulation (Fickentscher and Weiss, 2017). P lineage cells showed markedly different volume ratios, and divisions of ABar, EMS, MSa, MSp, Ca, and Cp were also significantly asymmetric. In contrast, E, MS, and C underwent almost perfectly symmetrical divisions. We applied the same scheme and criterion as the previous study (Fickentscher and Weiss, 2017, see Methods) and evaluated the asymmetry of the 27 cell divisions in 52 embryos (Figure 5). Cell divisions were deemed significantly asymmetric if the volume ratios of their daughters exceeded uncertainty levels, which is calculated as the sum of voxels comprising a layer around each cell (see Methods for details). A significant asymmetry was found in all P lineage cells, ABar, MSa, MSp, and Cp, all of which were also detected in the previous study. Especially, P lineage cells showed markedly different volume ratios, and the order of degree of asymmetry was consistent with the previous study. The divisions were almost equal in size in MSa, MSp, and C, congruous with the previous findings. In contrast, the asymmetry was not significant in EMS and Ca cells, which divided asymmetrically in the previous study. The discrepancy may be related to the definition of symmetry (see Discussion). A recent study that performed quantitative analysis of cell volume showed that divisions of EMS and Ca were symmetric (Guan et al., 2021), which is consistent with our results. Altogether, our results showed agreement with the previous study in 93% (25/27) of divisions.

While their analysis was limited to the divisions until the onset of gastrulation, our data permits the analysis beyond gastrulation. We applied the same analysis for 79 divisions, including the previous 27, in 35 embryos (Supplementary Figure S9). Surprisingly, asymmetry was more significant in Caa than in any of the P lineage cells. The median volume ratio was 2.9 in Caa. We manually checked some original images and confirmed that the segmentation results were correct. In addition, we found 12 new asymmetric divisions. These results demonstrate that our data can be used to reproduce the previous study and extend the study.

### 2.4 Variability of cell morphodynamics

**FIGURE 6:**
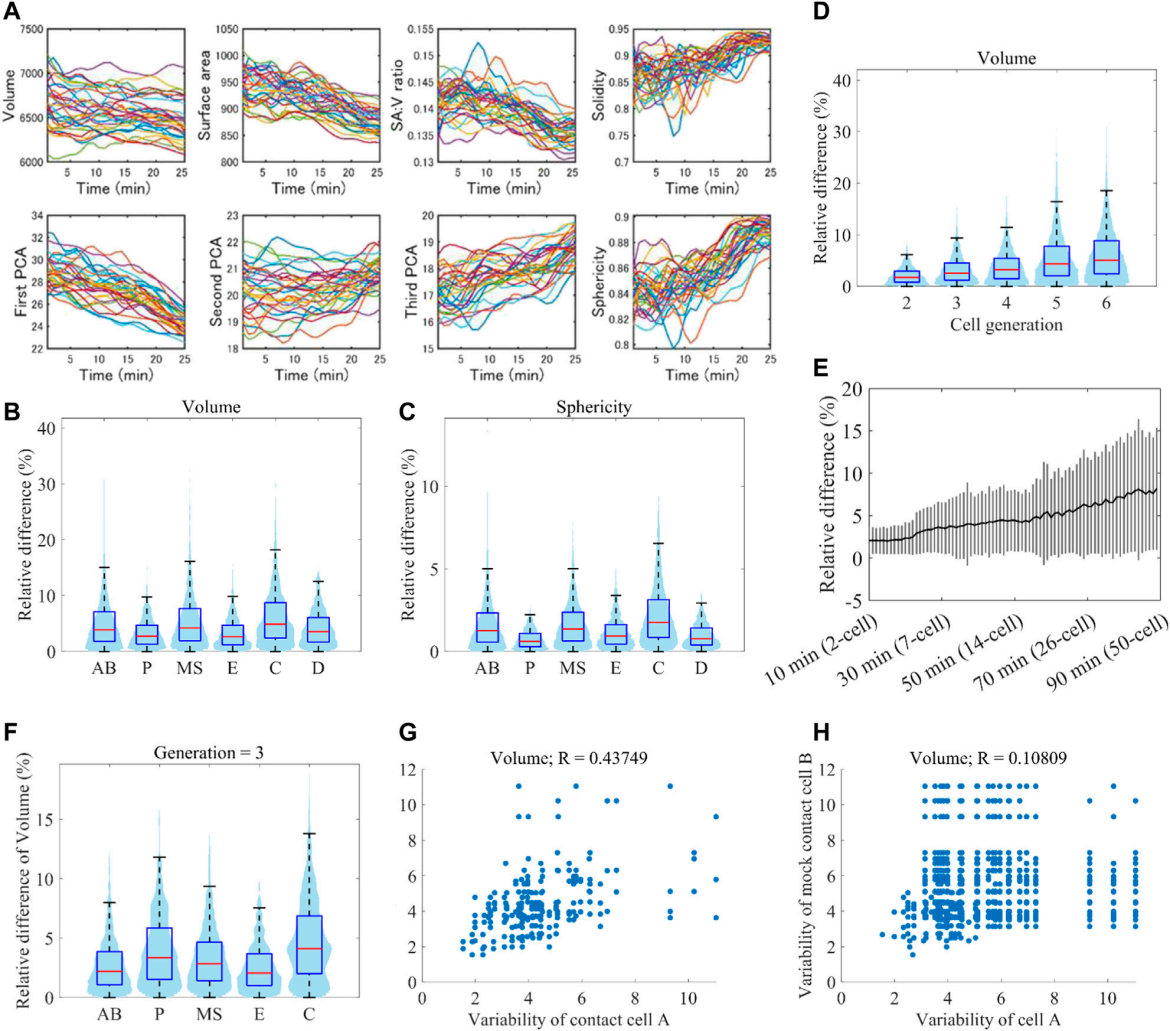
Reproducibility of morphodynamics **(A)** Spatiotemporally normalized single-cell feature dynamics in ABp cell. The dynamics of the 52 embryos are shown in different colors **(B and C)** Violin and box plots of the variability of volume **(B)** and sphericity **(C)** dynamics in each cell lineage. On each box, the central mark indicates the median, and the bottom and top edges indicate the 25th and 75th percentiles, respectively. The whiskers extend 1.5 times the interquartile range **(D)** Violin and box plots of the variability of volume dynamics in each cell generation **(E)** Increase of volume variability with time at single-cell resolution. Black line indicates the average of all cells at the time, and gray vertical lines indicate standard deviations **(F)** Violin and box plots of the variability of volume dynamics in each cell lineage at the cell generation 3 **(G and H)** Correlation of variability of volume dynamics between contact cells **(G)** and mock contact cells at the same cell generation **(H)**.

A previous study showed that cell position variability was lineage-specific (Li et al., 2019), raising the possibility that variability of cell morphodynamics is also lineage-specific. We normalized embryonic size and cell cycle length (Figure 6A) and measured the variability of eleven single-cell features between all pairwise combinations of the 32 embryos (Methods). The variability was larger in AB, MS, and C lineages than in P, D, and E lineages in every 11 features, indicating lineage specificity of the variability of morphodynamics (volume in Figure 6B, sphericity in Figure 6C, and all features in Supplementary Figure S10). The variabilities of AB, MS, and C lineages were significantly larger than at least either of those of P, D, and E (*p* < 0.01, Welch two-sample *t*-test). The previous study also found that the position variability dynamics showed a low-high-low pattern, where the variability increased with time until the mid-embryogenesis and decreased thereafter (Li et al., 2019). The increase was observed in the average dynamics of all cells and each cell level. As our data range is up to the mid-embryogenesis, corresponding to the increase phase, we examined whether the morphological variability increased with the development progression (volume in Figure 6D, all features in Supplementary Figure S11). As expected, the variability increased with cell generation. To check whether the increase was caused by an artifact of classifying the cells into the generations, we quantified the variability dynamics at single-cell resolution (volume in Figure 6E, all features in Supplementary Figure S12). Again, we observed a significant increase of the variability (between the initial and final time points, *p* < 0.01, Welch two-sample *t*-test), demonstrating the increase of morphological variability with the progression of embryogenesis.

Since each lineage is composed of different generations of cells, the lineage specificity may be caused by the mixture of different generations of cells. To check this, we compared the variability of the lineages at each cell generation (volume in Figure 6F). In each generation, we observed that specific lineages were more variable than the others. For example, in the cell generation 3, C was larger than AB, MS, and P in every feature (Supplementary Figures S13–S16). Hence the variability is lineage-specific even if we take the increase of variability with time into consideration.

The previous study also showed that position variability was more correlated between contact cells than between mock contact cells (Li et al., 2019). We examined whether the morphodynamics is also dependent on the cell-cell contact. We compared the correlation of morphodynamics variability between contact cells and mock contact cells (volume in Figure 6G (contact cells), 6H (mock cells), all features in Supplementary Figure S17). The correlation coefficient of volume dynamics between contact cells was 0.44, which is higher than that between mock contact cells (R = 0.11). In the other features, the correlation coefficients were also higher between contact cells than those between mock contact cells (Supplementary Figure S17), suggesting that the variability of morphodynamics is dependent on cell-cell contact.

These results show that the variability of morphodynamics is determined by multiple factors, including cell lineage, cell generation, and cell-cell contact.

### 2.5 Comparison with *P. mammillata*

**FIGURE 7:**
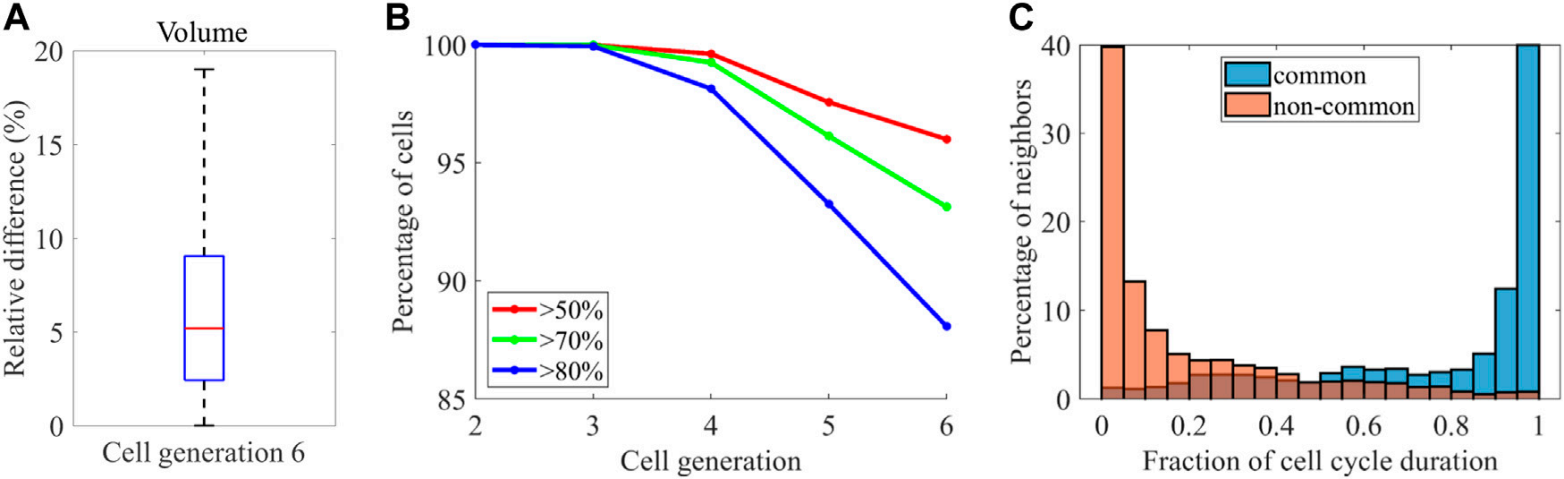
Comparison with *P. mammillata* **(A)** Variability of the median volume of the cell cycle across the embryos at the generation six **(B)** Percentage of cells at each cell generation showing conservation in neighborhood larger than the indicated thresholds **(C)** Histogram showing the distribution of contact duration of common neighbors (blue) and non-common neighbors (orange).

A previous study systematically quantified cell morphodynamics during ascidian *P. mammillata* embryogenesis, which displays a stereotypical pattern of cell orientations and divisions like *C. elegans* (Guignard et al., 2020). They compared cell position variability between *P. mammillata* and *C. elegans* and found that the variability was higher in *C. elegans*. The result raises the possibility of higher volume variability in *C. elegans*. Contrary, they reported that embryo size varied up to twofold, which is larger than *C. elegans* embryos [up to 1.42 times in our data). In addition, while the number of cells is invariant between embryos in *C. elegans*, it is not perfectly invariant (2% variation (Guignard et al., 2020)]. The facts raise the opposite possibility that volume variability is smaller in *C. elegans* embryos. While they quantified the cell volume, they could not compare the volume variability because there was no quantified volume data in *C. elegans*. As our data enables the comparison, we tested the controversial possibilities. We need to compare the variability at the same developmental stage because the variability increases with time in both animals (Figure 6D), [Li et al., 2019; Guignard et al., 2020)]. Although our data contains cell generations from two to six and the ascidian contains cell generations from seven to ten, the generation six in *C. elegans* corresponds to the generation seven in *P. mammillata* because the zygotic cell is the generation zero in *C. elegans* and the generation one in *P. mammillata*. We quantified cell volume variability determined in (Guignard et al., 2020) (Methods). The median variability of cells at this generation was 5.1% (Figure 7A), which is higher than *P. mammillata* (below 5%), which is consistent with the higher variability in cell positions (Guignard et al., 2020).

They also found high reproducibility of cell-cell contacts between embryos. We quantified the variability of cell-cell contact as defined in (Guignard et al., 2020) (see Methods for details) and found that the reproducibility of cell-cell contact decreased with cell generation (Figure 7B). At the cell generation six, 88% of cells shared at least 80% of neighboring cells, which is smaller than the reproducibility at the same generation in *P. mammillata* embryos (nearly 100% of cells shared at least 80% of neighboring cells). Similarly, 96% of cells shared at least 50% of neighboring cells, which is still smaller than the reproducibility at the same generation in *P. mammillata* embryos. Therefore, the cell-cell contact is less reproducible in *C. elegans*, which is consistent with the reproducibility of cell volume. In *P. mammillata*, contact with the neighbors lasted throughout the cell cycle (Guignard et al., 2020). Thus, stochastic cell neighbor exchanges were rare. We tested whether the cell neighbor exchanges were rare in *C. elegans*. The contact duration was quantified as the fraction of contacting period to the shorter cell cycle length of each contact pair (Figure 7C). More than half of contacts lasted more than 90% of the cell cycle in common neighbors shared across the embryos. Contrary, more than half of contacts lasted less than 10% of the cell cycle in non-common neighbors that were not shared by the embryos. Hence, stochastic cell neighbor exchanges are rare in *C. elegans*.

## 3 Discussion

We quantified cell morphodynamics in 52 *C. elegans* embryos from the two-cell stage to mid-gastrulation. We systematically analyzed extracted morphological features to obtain biological knowledge. The analysis of sphericity and volume dynamics showed that mitotic cell rounding occurred in all cells, whereas mitotic swelling occurred in most but not all cells. Among the exceptional cells (Supplementary Figure S7C), ABa and ABp showed characteristic sphericity dynamics (see Results). Ea and Ep are known to ingress inside the embryo at the beginning of gastrulation (Nance et al., 2005) and have significantly longer cell cycles than the other cells due to the introduction of a Gap phase (Edgar and McGhee, 1988). These characteristics might be related to the disappearance of mitotic swelling. In contrast, we could not find a reasonable explanation for the ABprppp. Although the segmentation accuracy of ABprppp, measured by the volume deviation between the adjacent time points, was the least among the 76 cell types, the same result was observed using only high-accuracy data. Hence, we consider that mitotic swelling does not occur in ABprppp. The sphericities at the end of metaphase varied from 0.80 to 0.90 (average, 0.85). There was a weak correlation between the sphericity at the initial and the final time points of the mitotic rounding (correlation coefficient, 0.33). However, the correlation was independent of the cell lineage, the developmental stage, and the cell volume (Supplementary Figures S6D–F). Therefore, the final sphericity may depend on other factors, such as the stiffness of the surrounding environment. In contrast, we found a correlation in the volume between the initial and final time points of the mitotic swelling (the Pearson correlation coefficient averaged for all cells was 0.73), which was more substantial than that (0.33) in sphericity. A correlation was also reported in the study that measured the volume change of cultured cells (Pearson correlation coefficient = 0.53, Son et al., 2015), supporting our results. On the other hand, we found that durations of mitotic rounding and swelling were consistent (7 min). Despite different cell cycle lengths, the duration is nearly equal for most of the cells. One reason is that cell cycle duration is likely elongated by S phase elongation at least before the 16-cell stage (Edgar and McGhee, 1988). Therefore, the duration of M phase is nearly equal between cells, making the time ranges of mitotic events equal.

In evaluating the reproducibility of cell-cell contact, we introduced the integral area of contact to minimize the influence of false detections. A straightforward way to remove false detection is a threshold-based method. Thus, detections whose contact areas are below the threshold are regarded as false positives. However, the detections are highly dependent on the threshold, which is not easy to decide reasonably. Indeed, a previous study that used thresholds for contact area and duration to remove false positive detections suffered from false negatives (Cao et al., 2020). In contrast, our integral area-based approach removes no detections, including false positives. Thus, no false negatives occur. False positives may be detected, but integral areas are usually very small because false detection rarely continues over multiple time points. The false positives can be manually checked. Indeed, we performed manual verification when classifying the contacts into three categories (Figure 4B). Hence, manual effort based on a well-established systematic analysis is one solution to manage imperfect systematic detections.

In the reproduction and extension of the previous study that investigated whether the division is asymmetric for 27 divisions during early embryogenesis s (Fickentscher and Weiss, 2017), our data showed agreements with the study in 25 divisions. Only two divisions were not significantly asymmetric in our data despite being significantly asymmetric in the previous study. The definition of uncertainty partially causes it. The uncertainty (see Methods and Fickentscher and Weiss, 2017) depends on image resolutions. The resolutions were larger in our data in both XY and Z. As a result, the uncertainty in our data was roughly double to triple of the previous study in each cell, which might reduce detection in this study. The analysis beyond gastrulation found additional asymmetric divisions and markedly different volume ratios between the daughters of Caa. The larger daughter Caaa produces four hypodermal cells, whereas the smaller daughter Caap produces one hypodermal cell, two neurons, and 1 cell death (Sulston et al., 1983). The high degree of asymmetry may reflect the descendant’s cell death because most cells undergoing cell death are generated as smaller daughters compared with their sisters by asymmetric divisions of their mothers (Hatzold and Conradt, 2008; Conradt et al., 2016). To check this hypothesis, we searched cells whose daughters differed by more than 50% in volume and found seven cells. We traced the lineages of their daughters and compared the numbers of descendants undergoing cell death. We found that six of the seven cases followed the hypothesis. Thus, smaller daughters had more descendants undergoing cell death. In the exceptional case, the same number of descendants underwent cell death. Therefore, cell death in descendants may be reflected in the high degree of volume asymmetry. The asymmetric division has been suggested to have a functional link to apoptosis (Hatzold and Conradt, 2008). Our results show that smaller daughters had more descendants undergoing cell death. This raises the possibility that asymmetric divisions at several rounds of cell divisions ahead have a functional link to apoptosis. However, the small number of tested samples and an exception require additional investigations to verify this hypothesis.

The variability of cell position was shown to be highly deterministic and determined by cell lineage coupled to diverse developmental properties of cells (Li et al., 2019). In addition, the position variability was shown to increase with time until the mid-embryogenesis and decreased thereafter. However, variability has not been clarified for cell morphology because of a lack of data. We showed that the variability of cell morphodynamics is also specific to the cell lineage. The specificity was observed in every morphological feature. The variability was also dependent on the cell-cell contact, indicating that the intrinsic and extrinsic factors affect the variability. We also found that morphological variability increased with time until mid-embryogenesis. As our data traced the embryogenesis until this stage, it is unclear whether the variability decreases thereafter, just as the position variability was. In future work, it is important to elucidate this question with more thorough data.

We compared the variabilities of morphodynamics and cell-cell contact with those of ascidian *P. mammillata*, which displays a stereotypical pattern of cell orientations and divisions during embryogenesis like *C. elegans*. The cell position variability was shown to be higher in *C. elegans* (Guignard et al., 2020). Consistent with this, we found that variabilities of cell volume and cell-cell contact were also higher in *C. elegans*. The results are intriguing when considering the facts that the number of cells is perfectly invariant in *C. elegans* whereas quasi-invariant in *P. mammillata,* and the embryo size difference was up to 1.4 times in *C. elegans* whereas up to twofold in *P. mammillata*. It may be related to the difference in the conservation of embryo geometry between nematodes and ascidians. The geometry of ascidians is essentially unchanged from the emergence of the group around 400 million years ago (Lemaire, 2011; Delsuc et al., 2018), whereas it has been changed in *C. elegans* (Goldstein, 2001). However, we just compared the variability at 1 cell generation. A more thorough comparison is needed.

## 4 Methods

### 4.1 Imaging

Sample preparation and imaging methods are described in (Azuma and Onami, 2017), except that a time interval of 30 s was used. The number of focal planes differed in each embryo. We recorded 52 embryos for 2 hours from the two-cell stage. We confirmed that all embryos hatched.

### 4.2 The network architecture of restworm

Restworm was developed by modifying the U-Net (Ronneberger et al., 2015) model. We introduced batch normalization and Leaky ReLU at each down- and up-sampling step. The convolution filter size was changed to 3 from 5. The filter size of max-pooling was changed to 2 from 4. The code and detailed network architecture are available at https://github.com/bioimage-informatics/restworm.

### 4.3 Cell lineage assignment

The cell lineage assignment method uses publicly available annotated data (Richards et al., 2013) as a reference and annotates all detected nuclei. A target data that is a set of nuclear coordinates at the final time point was taken. Reference data was selected from the annotated data as a set of nuclear coordinates with the same number of nuclei as the target data. If there were multiple such data, all of them were selected one by one. Principal component analysis (PCA) was applied to each target and reference embryo and mapped to each PC coordinate system. Then, the target embryo was rotated around the first PC axis at two-degree intervals and slightly swung along the other PC axes. In each step, the target embryo was scaled to fit in size to the reference embryo by multiplying a scaling factor to equalize the variance of cell positions in each axis and was applied the matching function, which measures the distance for every cell pair between the target and reference, and the distance was used as “cost” for matching them. The minimum cost matching was found using the Hungarian algorithm (Munkres, 1957). The matching was calculated for every step, and the cost was recorded. Finally, the matching with the minimum cost was selected as the annotation for the target data. By using the annotated cells at the final time point, the cells at earlier time points can be successively annotated by tracing back the lineage. If the annotation at the final time point is accurate, all cells can be accurately annotated until the two-cell stage. If it fails on the way, the annotation at the final time point is not accurate. In this case, a set of nuclear coordinates at one time point earlier than the final time point was used as the target data and annotation. The process was repeated until the tracing was successful.

### 4.4 Collection of the time lag between nucleus and cell divisions

We determined the end of cell divisions when the cell membrane completely encloses the cell. We estimated the timing by a support vector machine with eleven features, such as the intensity between two divided nuclei in the membrane image (Supplementary Table S4). The training data was manually created for 147 cell divisions in six embryos. The error rate was 1.7% in the 5-fold cross-validation.

### 4.5 Normalization of embryonic size and cell cycle length

We observed variations in absolute values of the features, especially those that were size-related, such as volume and surface area (Supplementary Figure S4). Embryo sizes are known to vary under identical conditions (Moore et al., 2013; Richards et al., 2013; Insley and Shaham, 2018). The ratio between the minimal and maximal volume was 1.42 in our data. To minimize these variabilities, we scaled each segmented embryo linearly to approximate its volume to the average of all the embryos. Our membrane segmentation method (BCOMS2) segments embryonic regions before membrane segmentation. Embryo volume was calculated at each time point by summing all the pixels of the embryonic region. Since the embryonic volume changes slightly throughout development, the median volume was used as the volume of the embryo. Then, an average embryonic volume was calculated from all the embryos. Each embryo image and each segmentation result were linearly expanded or shrunk to approximate its volume to the average volume.

We also observed deviations in the feature dynamics in the temporal direction. One possible source of the deviations is the differences in developmental rates between the embryos. The developmental rates vary even if the imaging conditions are consistent (Schnabel et al., 1997; Bao et al., 2008). Indeed, the ratio between the longest and shortest cell cycle length ranged from 1.14 to 1.40, depending on the cell type. Cell cycle lengths of a given cell vary between the embryos due to differences in developmental rates and fluctuations of division timings. The lengths ranged from 12 to 49.5 min for all the cells in the embryos, and the average was 25.2 min. Therefore, we normalized the temporal lengths of the feature dynamics by spline interpolation to make their length 25 min (50 time points).

### 4.6 Volume ratios of sister cells

The evaluation of cell volume and the significance of asymmetry was performed according to the method described previously (Fickentscher and Weiss, 2017). The median volume across the cell cycle was used as the volume. Cell divisions were deemed significantly asymmetric if the volume ratios of their daughters exceeded uncertainty levels, which arose solely from segmentation errors. In short, the uncertainty is calculated as the sum of voxels comprising a layer around each cell.

### 4.7 Measurement of morphodynamics variability

We used the metric used in a previous study (Guignard et al., 2020) for the morphodynamics variability. As the metric was defined for scalar values, we modified it for vectors. The variability v of two n length feature dynamics $f_{a}\left(t\right)$ and $f_{b}\left(t\right)$ was given as:

$$v=\frac{\overset{n}{\underset{t=1}{\sum }}\frac{\left|f_{a}\left(t\right)-f_{b}\left(t\right)\right|}{f_{a}\left(t\right)+f_{b}\left(t\right)}}{n}$$ (1)

The interval of v is $\left[0,1\right.)$ , which is 0 for perfectly consistent feature dynamics and one for approaching very different dynamics.

### 4.8 Metrics for comparison with *Phallusia mammillata*

The variability $M_{v}$ of cell *A* with a median volume $V_{A}$ across the cell cycle and cell *B* with a median volume $V_{B}$ across the cell cycle was given as:

$$M_{v}=\frac{\left|V_{A}-V_{B}\right|}{V_{A}+V_{B}}$$ (2)

The interval of $M_{v}$ is $\left[0,1\right.)$ , which is 0 for perfectly consistent feature dynamics and one for approaching very different dynamics.

The metric for the variability of cell-cell contact is described in detail in a previous study (Guignard et al., 2020). In short, For each cell, contacts with a contact area less than 5% of the cell’s surface area were removed as noise. Then, the reproducibility of neighbors between two embryos was given by the high value across the cell cycle of the shared neighbors in neighbors of two embryos. This metric gives values in the interval [0, 1]. A value of one means the 2 cells have exactly the same neighbors at any time in the cell cycle. A value of 0 means no common neighbors throughout the cell cycle.

## Data Availability

The datasets presented in this study can be found in online repositories. The names of the repository/repositories and accession number(s) can be found below: https://doi.org/10.24631/ssbd.repos.2022.06.236
