# Supplementary material for: Systematic analysis of cell morphodynamics in C. elegans early embryogenesis
Source: Front Bioinform. 2023 Mar 21;3:1082531. doi: 10.3389/fbinf.2023.1082531 (PMC10070942; doi:10.3389/fbinf.2023.1082531)
Supplement: Supplementary file 2 [file DataSheet1.PDF]

Supplementary Material

1 Supplementary Figures and Tables

1.1 Supplementary Figures

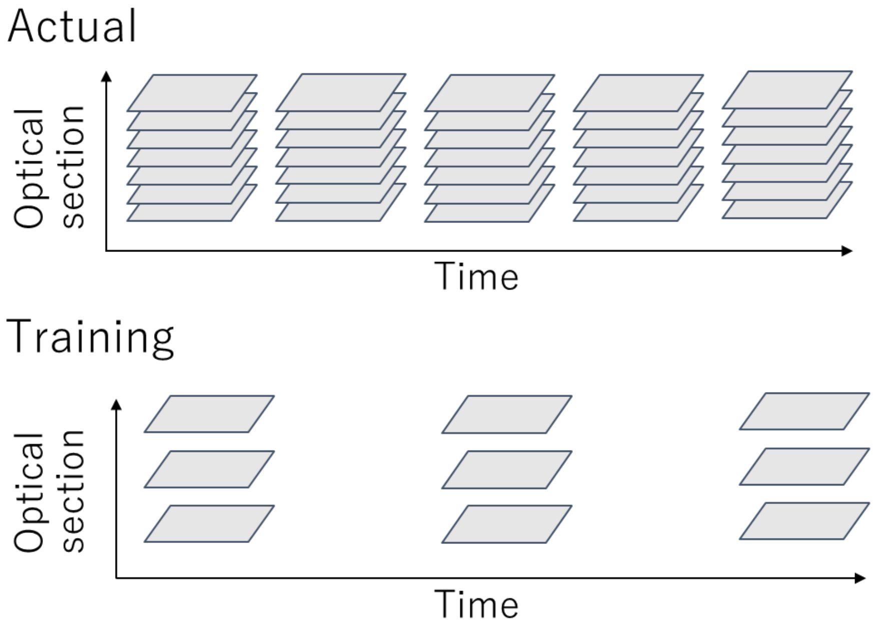

Supplementary Figure 1: Data sampling in the image restoration.

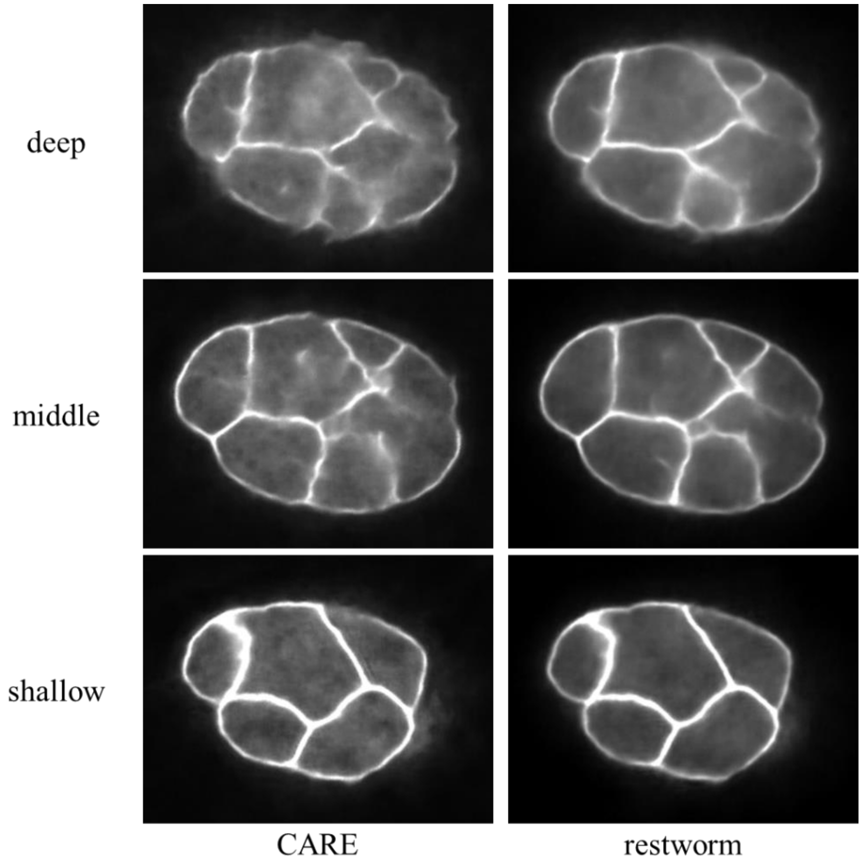

Supplementary Figure 2: Comparison of image restoration results.

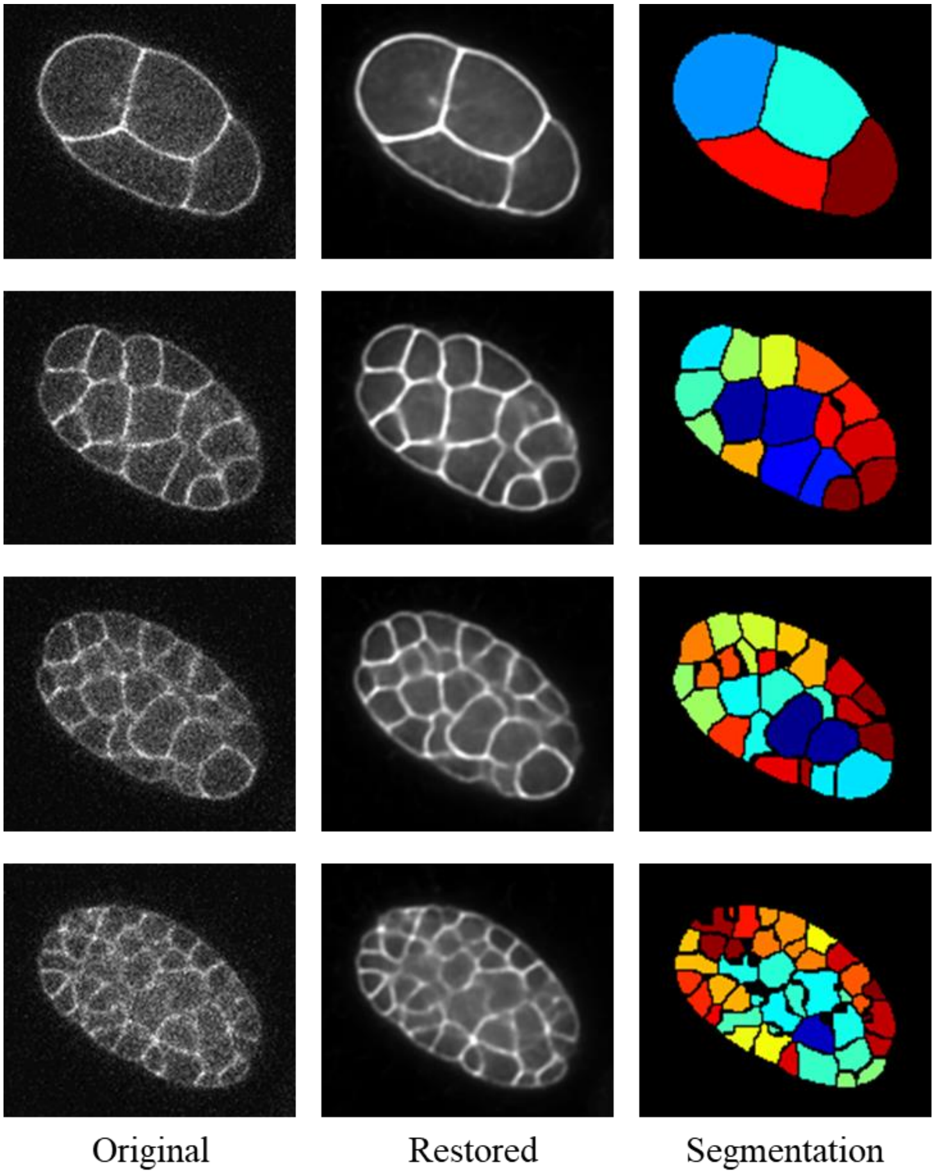

Supplementary Figure 3: Image restoration and segmentation results.

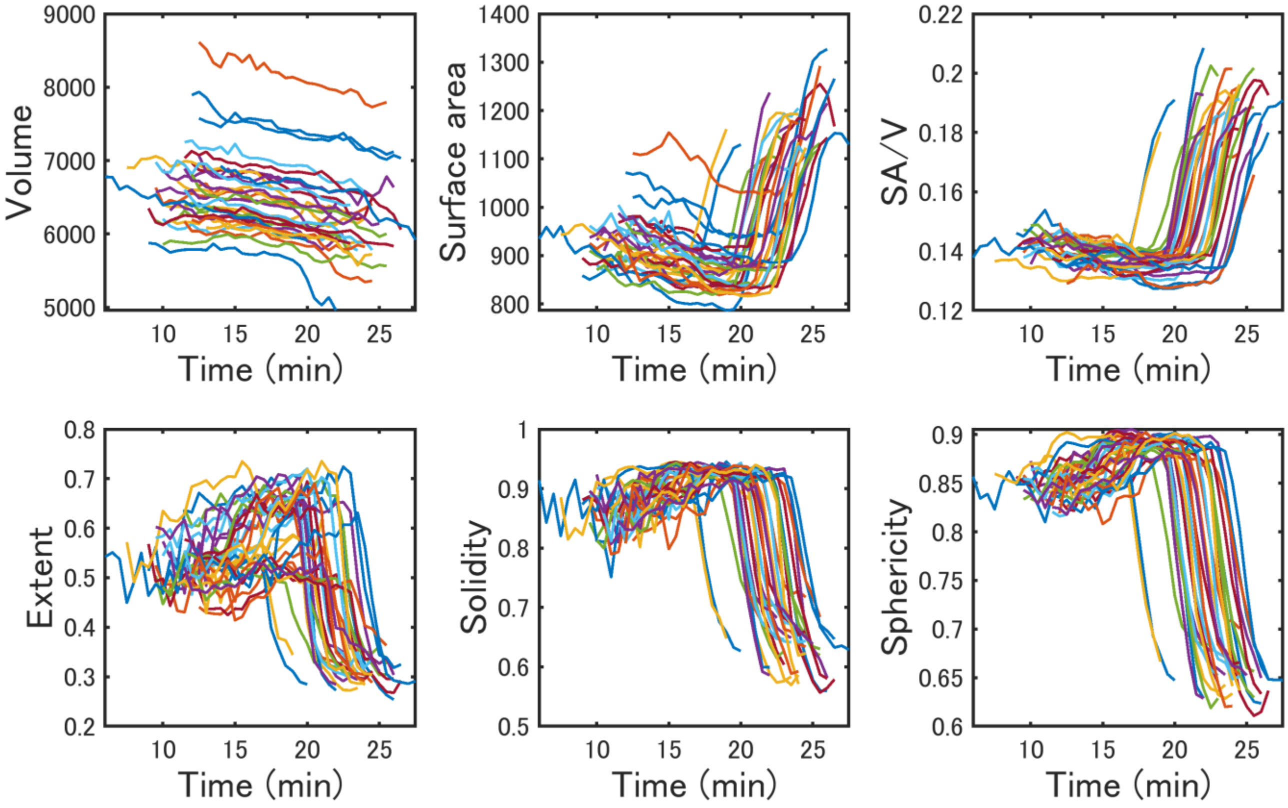

Supplementary Figure 4: Dynamics of single-cell features in ABp cell.

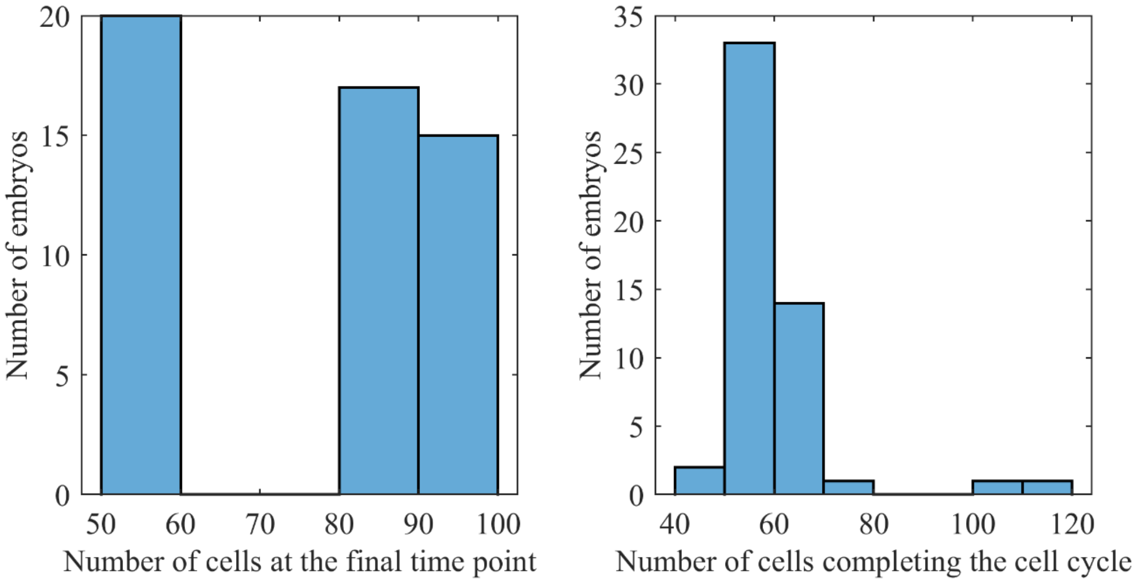

Supplementary Figure 5: Statistics of the cells and embryos.

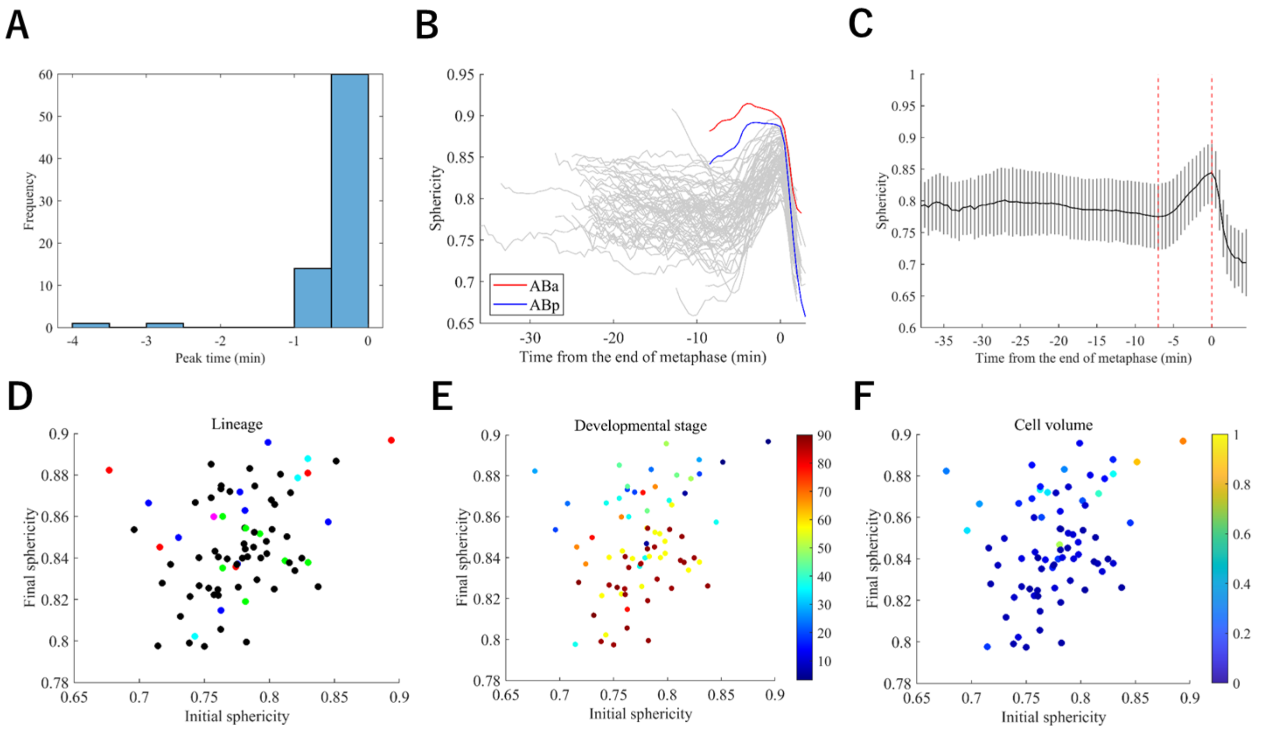

Supplementary Figure 6: Figures related to the mitotic rounding analysis.

(A) The distribution of sphericity peak times. (B) The sphericity dynamics of ABa and ABp. (C) The sphericity dynamics averaged over all cell types. Red vertical dotted lines indicate the initial and end of mitotic rounding. (D-F) Correlation between the initial and the final time points of the mitotic rounding colored by cell lineage (C), developmental stage (birth time) (E), and normalized cell volume (F).

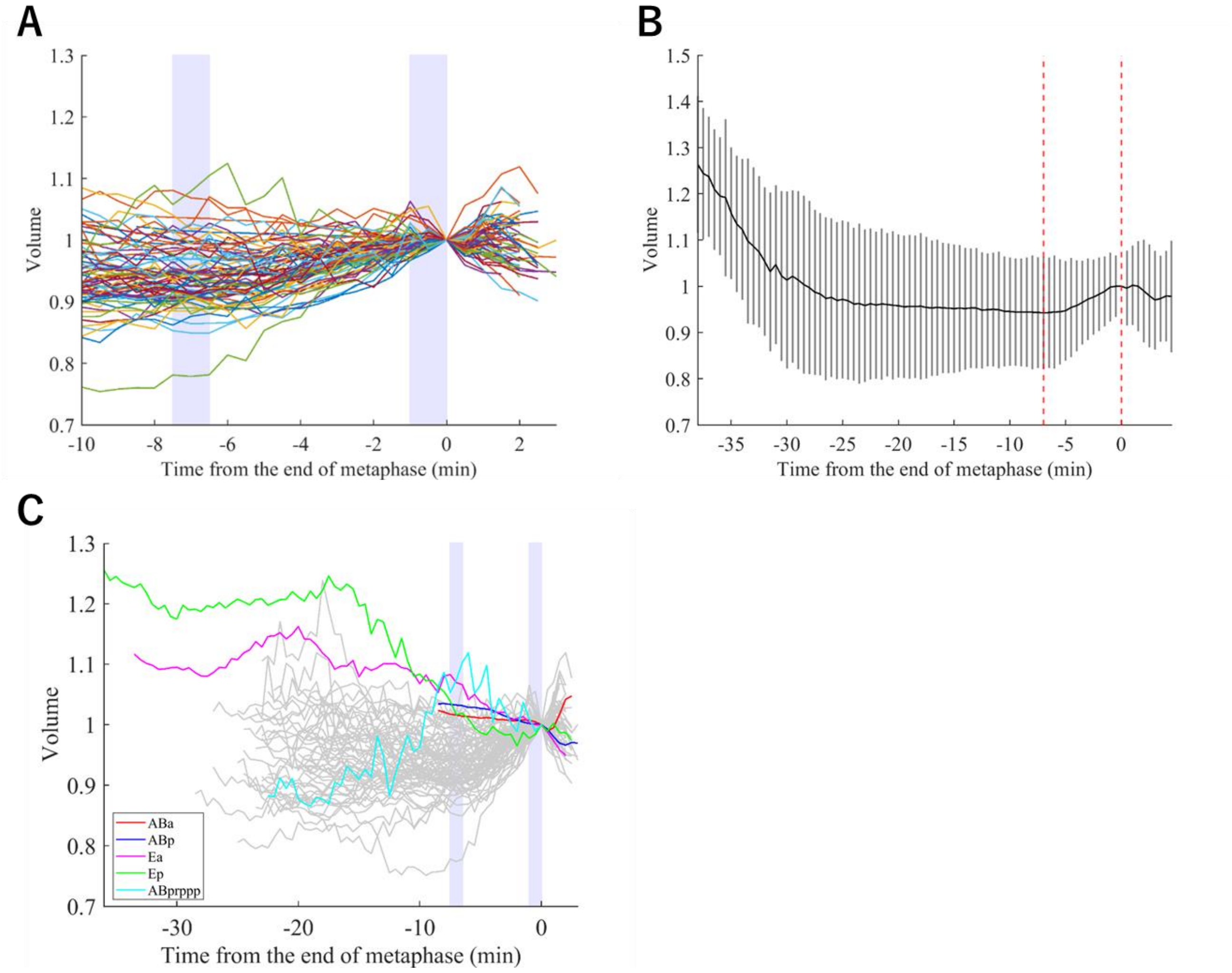

Supplementary Figure 7: Figures related to the mitotic swelling analysis.

(A) The cell volume dynamics around the period of the mitotic rounding. (B) The cell volume dynamics averaged over all cell types. Gray vertical lines indicate standard deviations. Red vertical dotted lines indicate the initial and end of mitotic rounding. (C) The volume dynamics of cells that did not show the mitotic swelling.

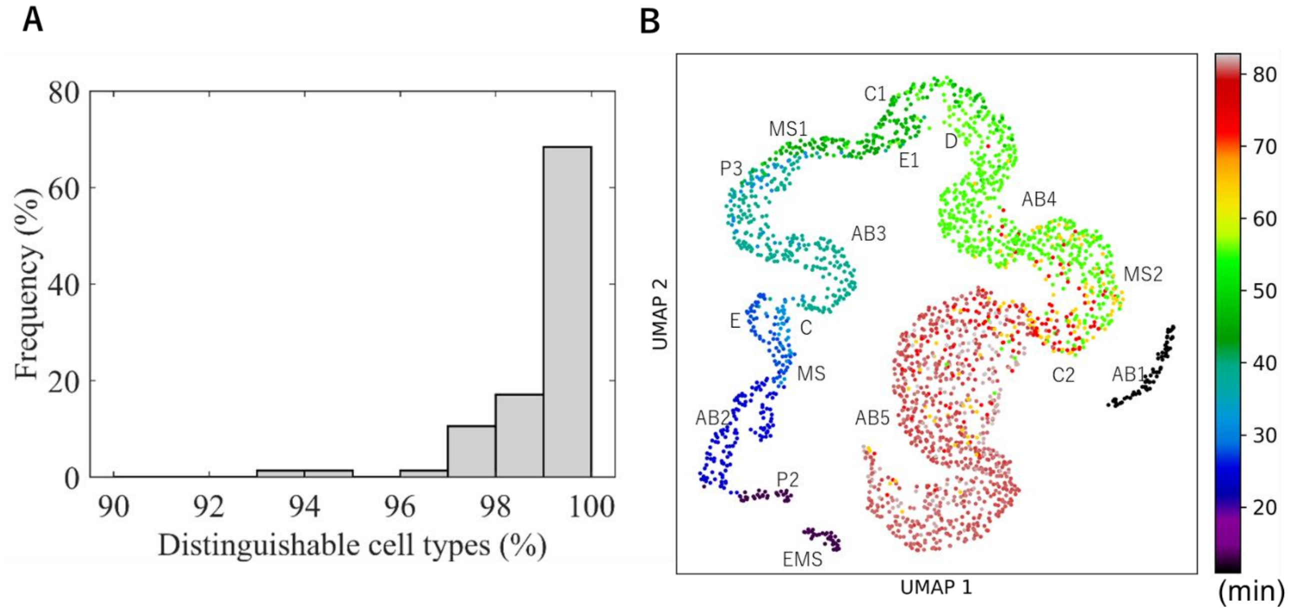

Supplementary Figure 8: The uniqueness of cell morphodynamics.

(A) Frequency of the number of cell types that can be distinguished from the indicated fraction of the other cell types. (B) Umap projection colored by birth time.

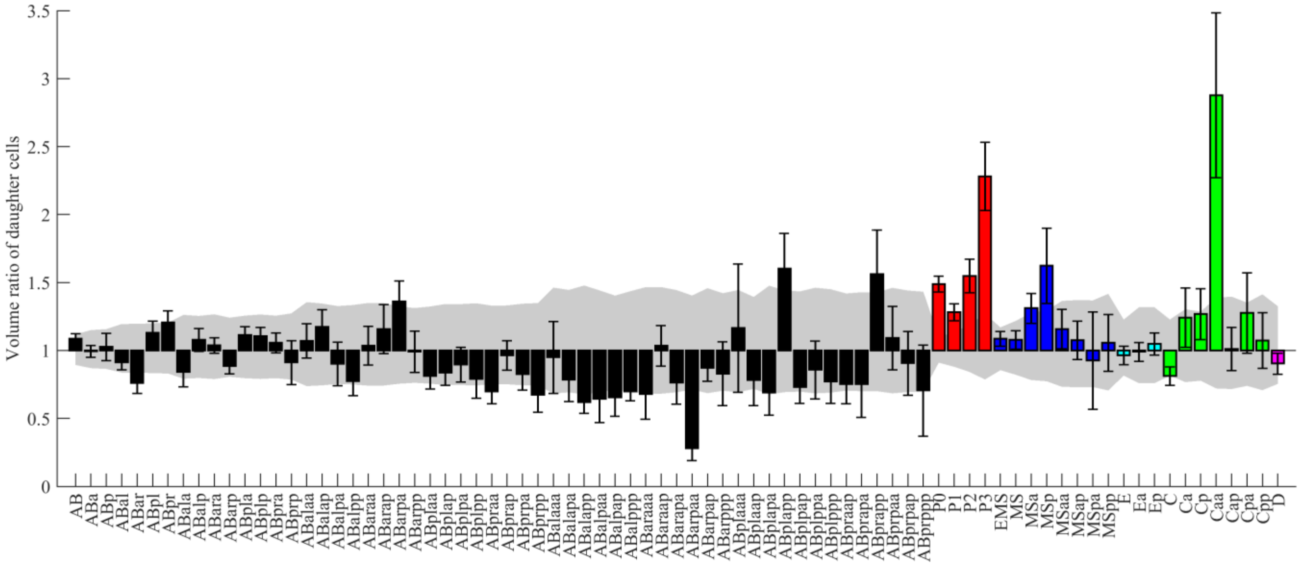

Supplementary Figure 9: Volume asymmetry of all cell divisions in our data.

Volume ratios of daughter cells emerging from the named mother cell. The design of the graph is the same as in Figure 1A. The medians of the 52 embryos are shown with error bars indicating standard deviations. Colors indicate cell lineages. The gray region indicates the volume-dependent level of uncertainty (see Methods for details).

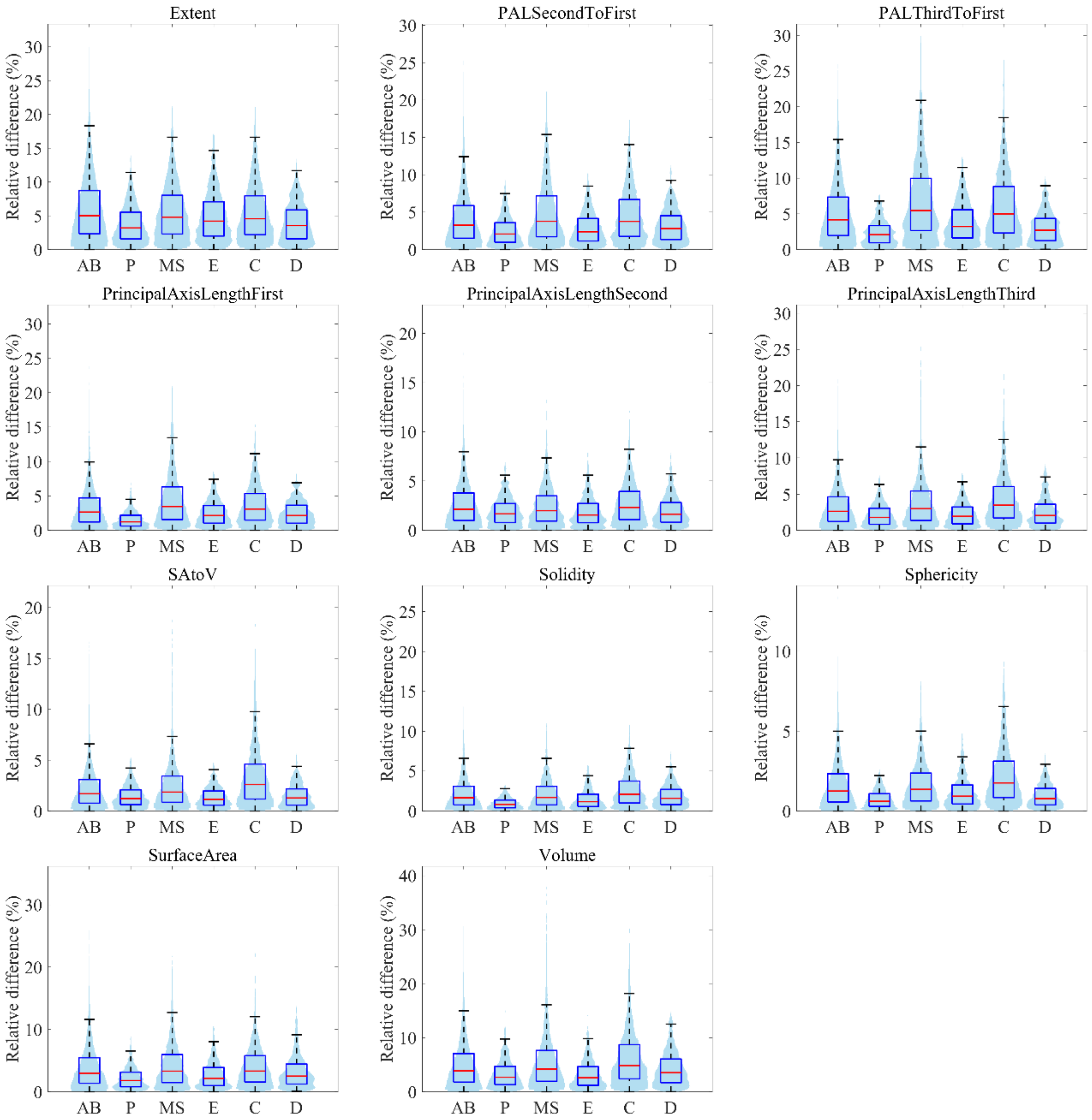

Supplementary Figure 10: Variability of morphodynamics in each cell lineage.

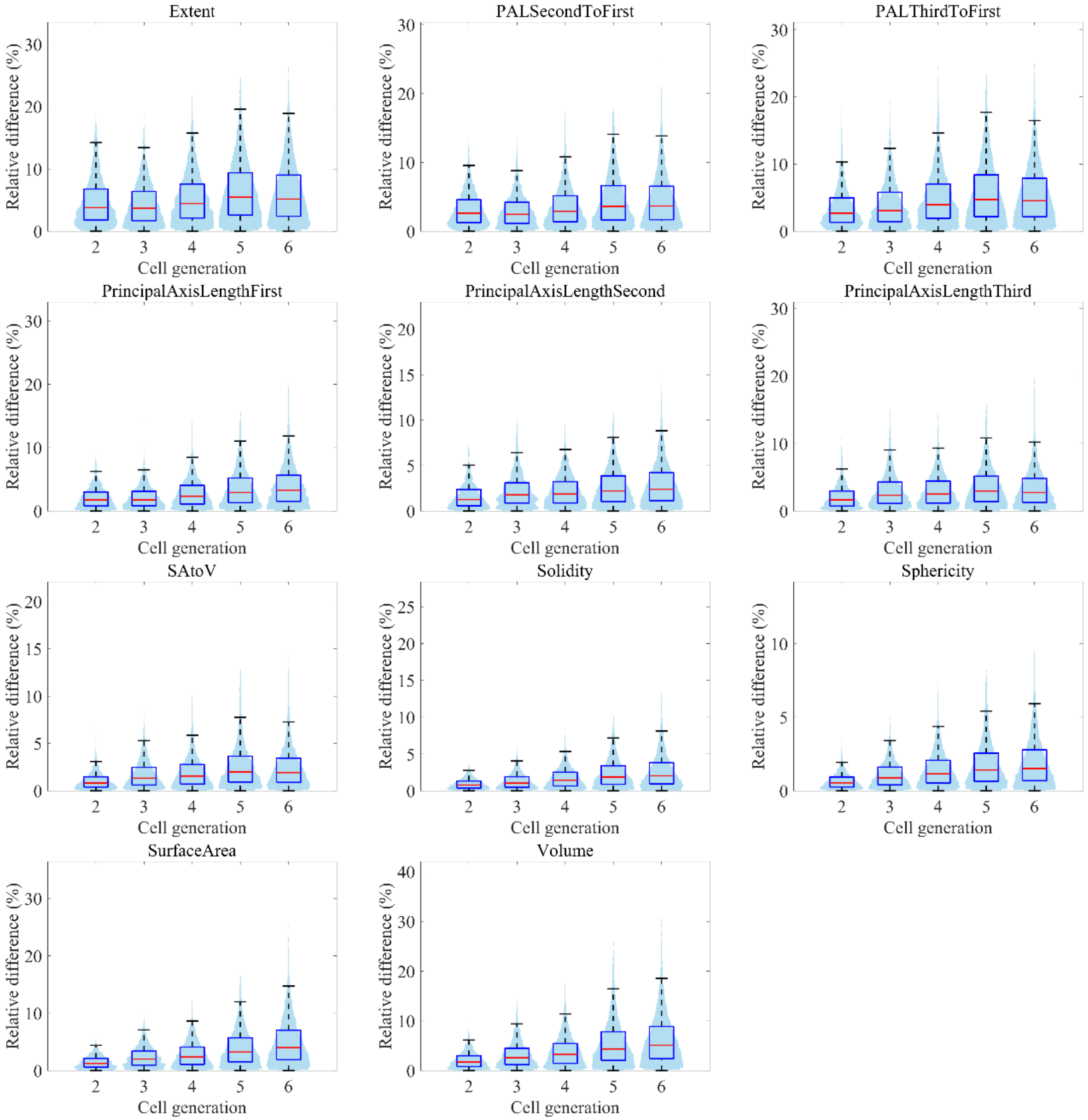

Supplementary Figure 11: Variability of morphodynamics in each cell generation.

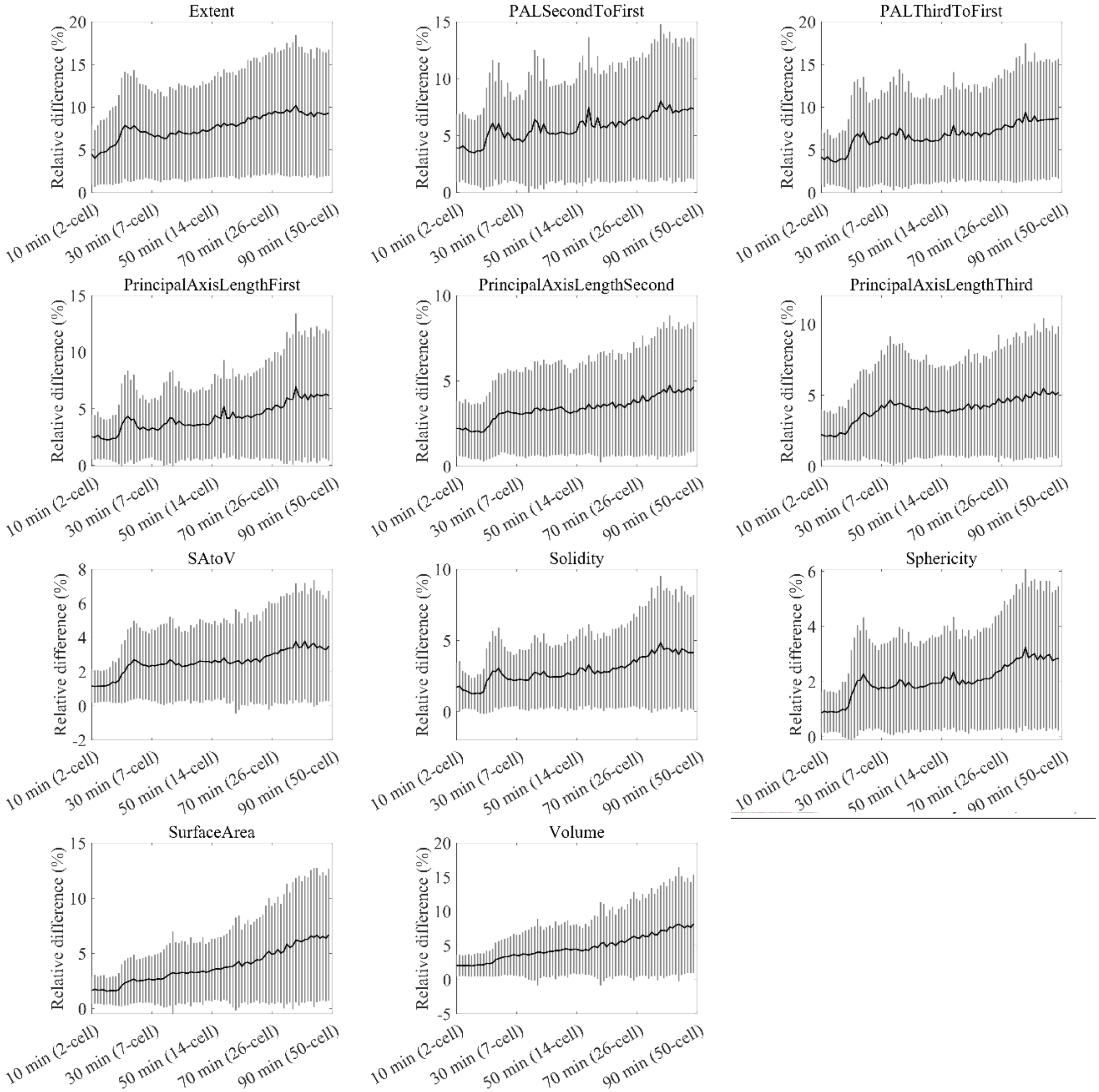

Supplementary Figure 12: Increase of morphological variability with time in single-cell resolution.

Black line indicates the average of all cells at the time and gray vertical lines indicate standard deviations.

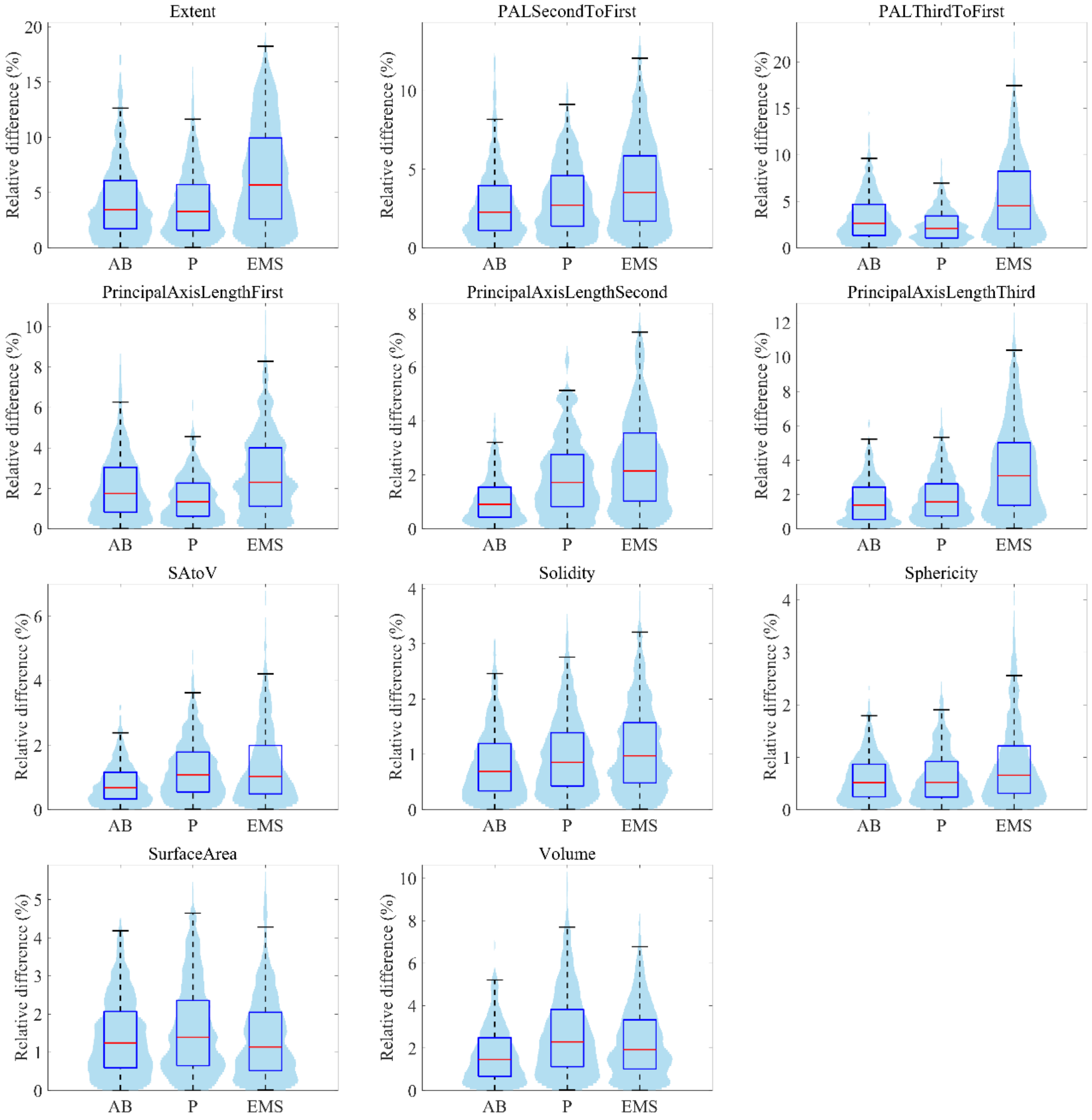

Supplementary Figure 13: Variability of morphodynamics in cell generation 2.

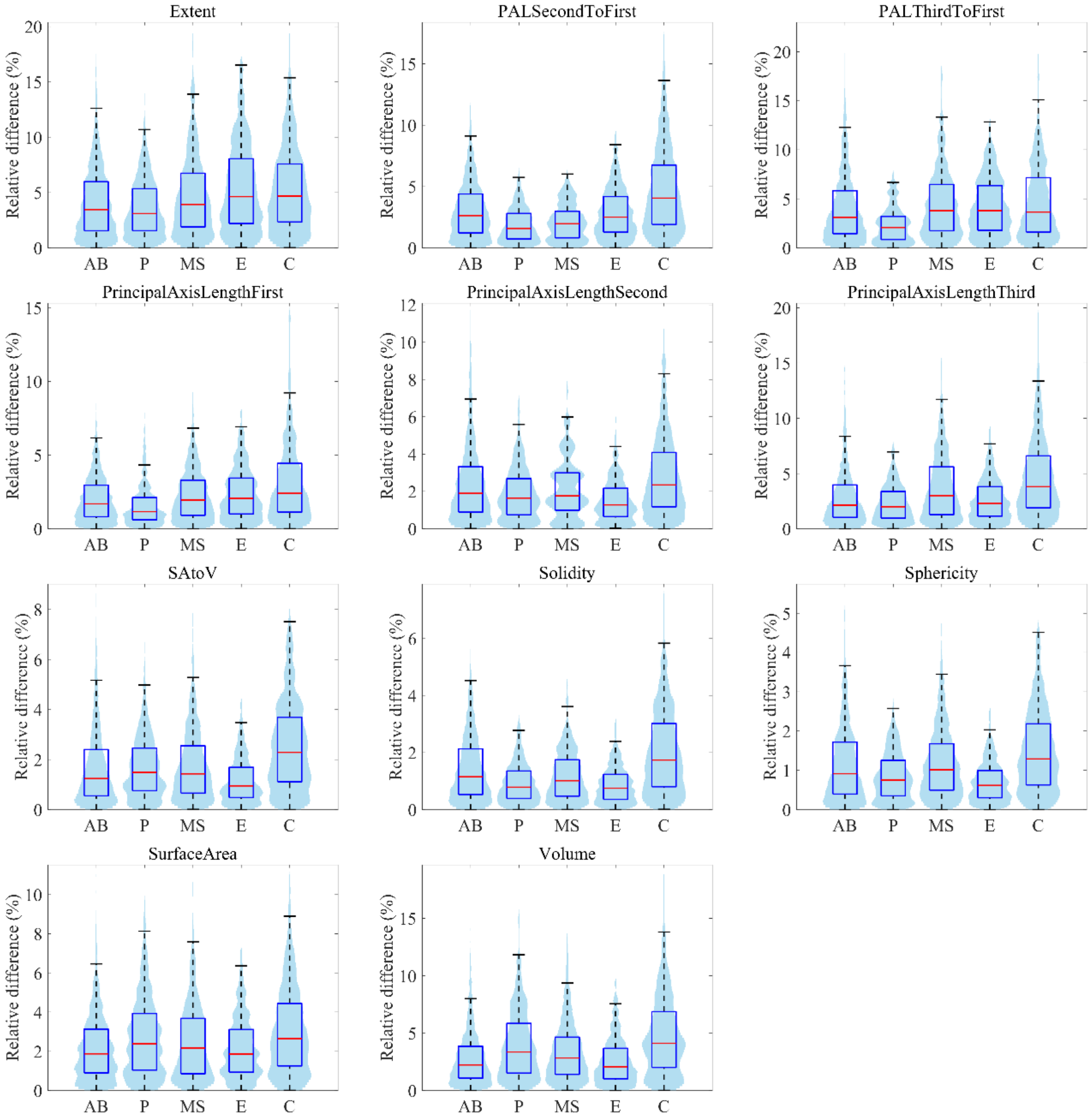

Supplementary Figure 14: Variability of morphodynamics in cell generation 3.

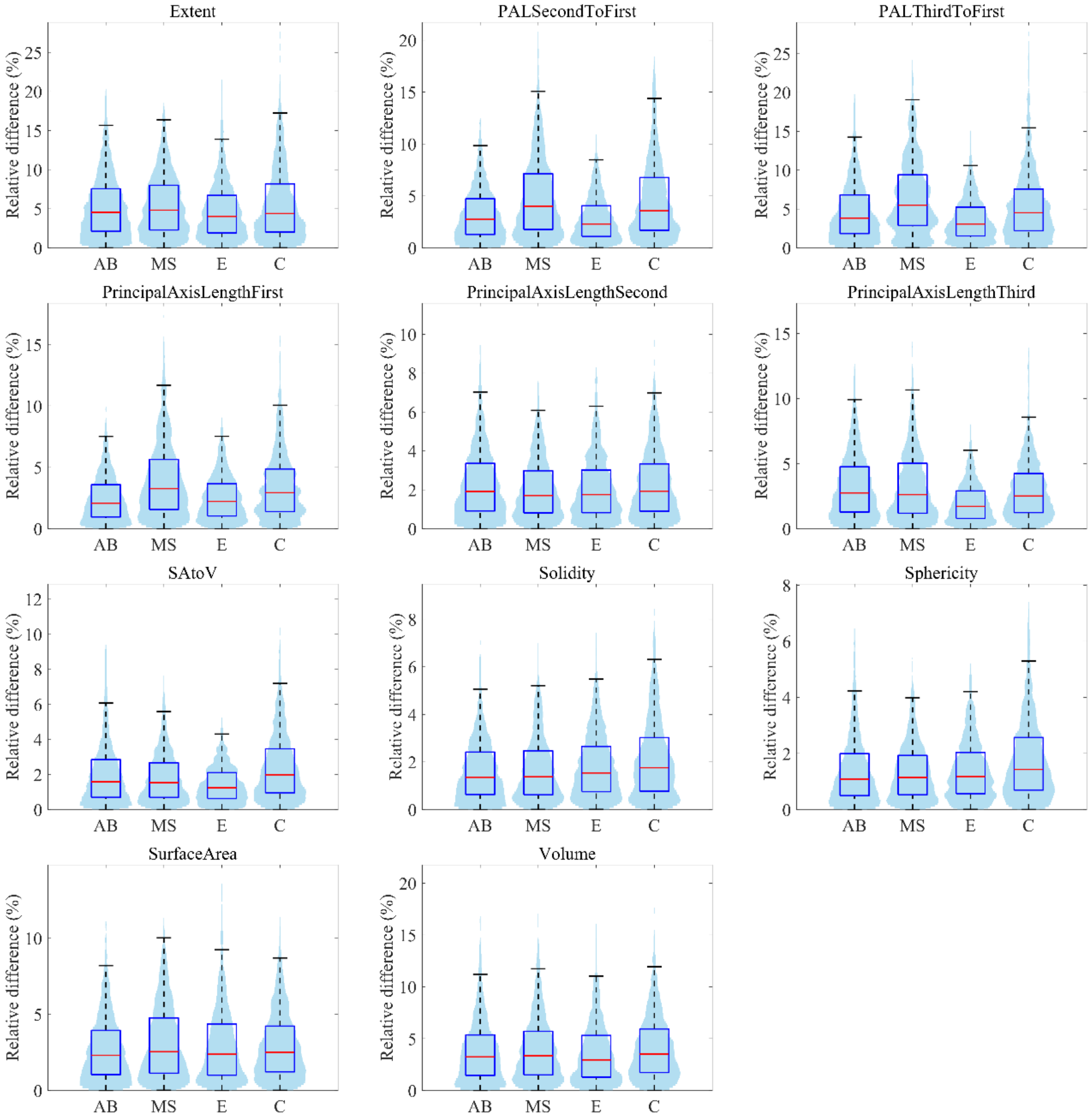

Supplementary Figure 15: Variability of morphodynamics in cell generation 4.

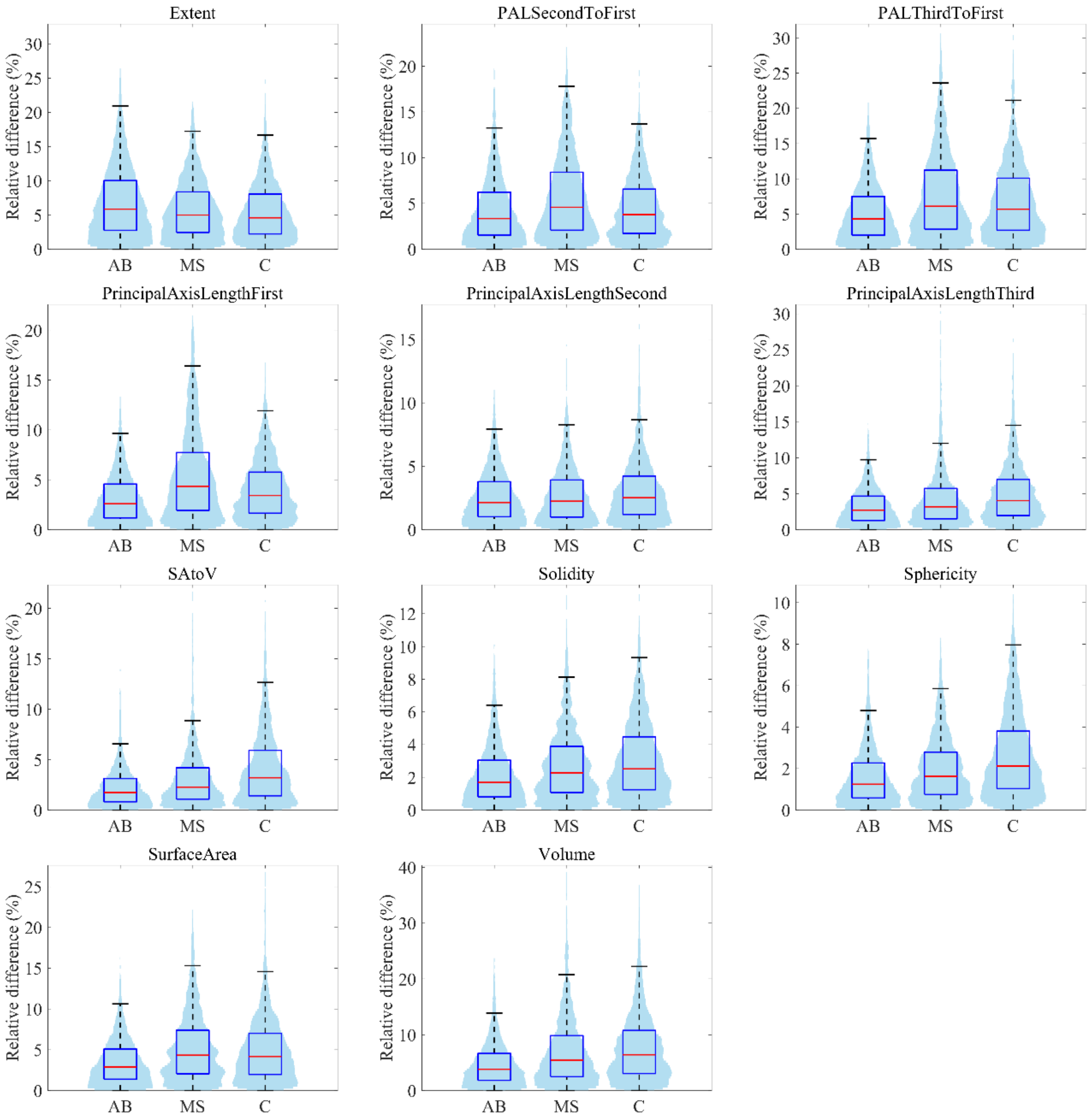

Supplementary Figure 16: Variability of morphodynamics in cell generation 5.

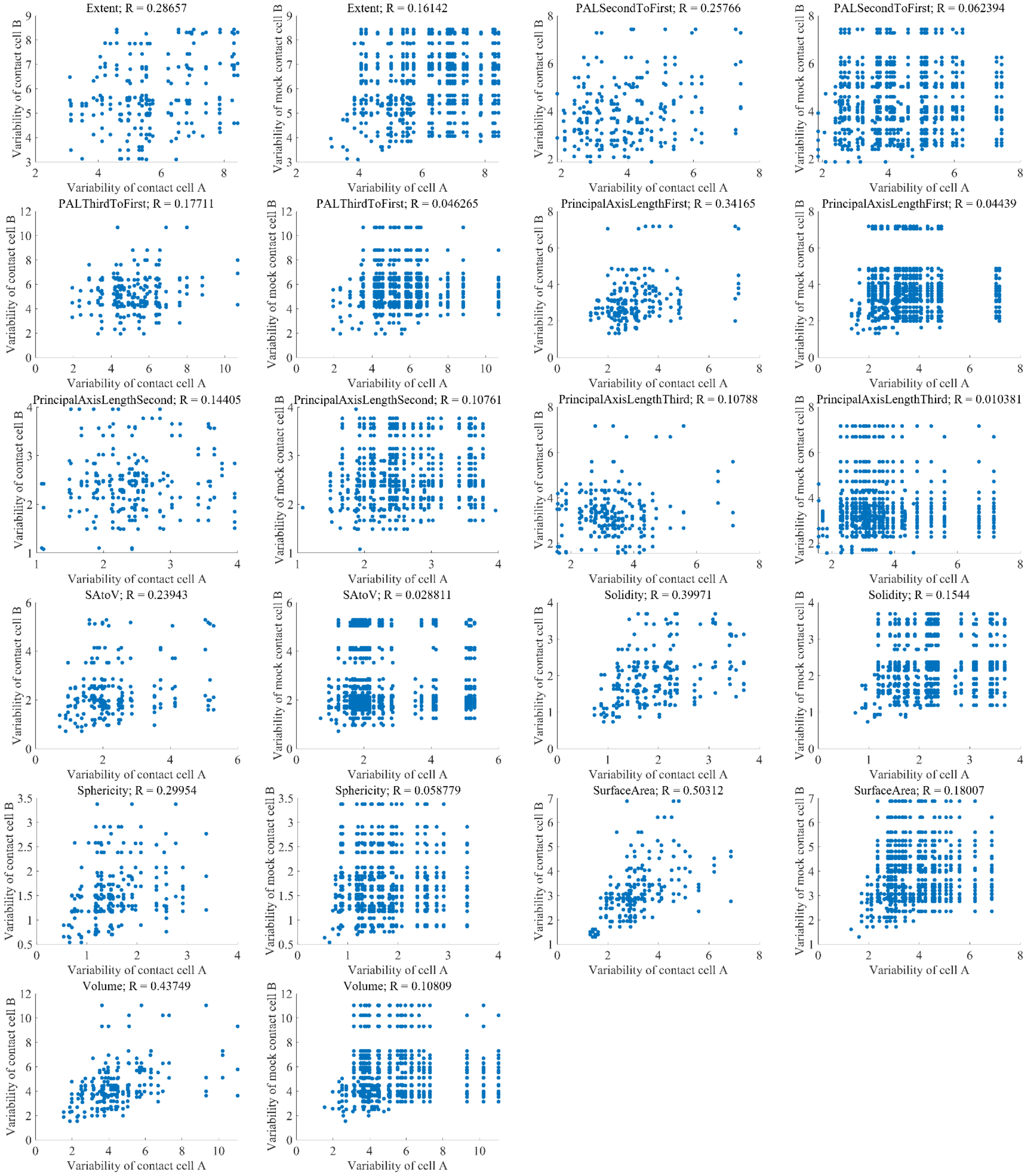

Supplementary Figure 17: Correlation coefficient of morphodynamics between contact cells or mock cells.

1.2 Supplementary Tables

Supplementary Table 1: Classification of the features.

Category	Feature name	Details
Single-cell	Volume	The number of voxels multiplied by the resolution
	Surface area	The number of voxels on the surface multiplied by the resolution
	SA/V	The surface area divided by the volume
	Solidity	The volume of the region divided by the volume of the smallest convex polygon that can contain the region
	Extent	The volume of the region divided by the volume of the smallest cuboid containing the region
	Sphericity	The extent to which the shape of the region resembles the perfect sphere
	Long axis	Length of the first PCA axis of the ellipsoid that has the same normalized second central moments as the region
	Middle axis	Length of the second PCA axis of the ellipsoid that has the same normalized second central moments as the region
	Short axis	Length of the third PCA axis of the ellipsoid that has the same normalized second central moments as the region
	Middle/Long	Middle axis divided by Long axis
	Short/Long	Short axis divided by Long axis
Inter-cell	Contact area	The number of voxels on the contact between the two cells
	Contact duration	The number of time points during which the contact is formed

Supplementary Table 2: Cell-cell interactions used in this study and analysis results.

Cell-cell interaction	Cell1	Cell2	Reproducibility	Integral area (μm^2)	Duration	Mean area (μm^2)	#Embryos	Reference
1st Notch	ABp	P2	100	8842	23	385	52	(Priess, 2005)
2nd Notch	MS	ABalp	100	3086	12	263	52	(Priess, 2005)
2nd Notch	MS	ABara	100	1338	11	120	52	(Priess, 2005)
3rd Notch	ABplaaa	ABalapa	100	4198	54	78	52	(Priess, 2005; Chen et al., 2018)
3rd Notch	ABplaaa	ABalapp	100	3038	54	57	52	(Priess, 2005; Chen et al., 2018)
4th Notch	MSapa	ABplpapp	100	782	17	44	32	(Priess, 2005; Chen et al., 2018)
4th Notch	MSapp	ABplpapp	100	1073	18	63	32	(Priess, 2005; Chen et al., 2018)
5th Notch*	ABplpppp	MSappp	100	91	2	46	2	(Priess, 2005; Chen et al., 2018)
Wnt	EMS	P2	100	8077	29	276	52	(Eisenmann, 2005)
Wnt	ABar	C	100	1272	14	94	52	(Walston et al., 2004)

* The cell cycle does not complete

Supplementary Table 3: Manual annotation results of variable contacts.

Cell1	Cell2	Integral area (μm^2)	Duration (time point)	Area(μm^2)	Annotation*
ABa	P2	12.8	1.3	11.2	FP
ABar	ABplp	32.5	1.2	26.1	VCA
P3	ABarp	73.3	1.0	73.3	FP
ABala	ABplap	25.6	2.2	10.8	VCA
ABalp	Ep	40.3	1.0	40.3	FP
ABara	ABalpp	0.8	1.0	0.8	VCA
ABarp	MSa	333.9	16.0	17.7	VCA
ABpra	ABplap	4.8	2.0	2.4	VCA
ABprp	ABarpp	44.1	2.0	17.2	VCA
MSa	ABprpa	39.4	1.5	30.1	FP
MSp	D	20.8	1.0	20.8	FP
Ea	Cp	482.4	29.7	12.1	VCA
Ea	ABaraa	28.0	1.5	17.2	FP
Ea	ABarpp	514.1	16.7	24.6	VCA
Ca	MSap	127.7	9.8	11.4	VCA
ABplap	ABprpa	10.8	1.0	10.8	FP
ABalaa	ABarap	49.9	1.8	23.0	FP
ABalpa	MSap	354.3	10.8	38.8	VCA
ABalpp	ABarpa	68.3	5.6	10.0	VCA
ABarpa	MSaa	258.3	12.3	15.8	VCA
ABarpp	MSpp	600.5	24.5	19.8	VCA
ABprpa	MSap	53.6	2.6	14.9	FP
ABprpp	MSpa	27.8	2.0	14.8	FP

*VCA: variability in cell arrangement, FP: false positive

Supplementary Table 4: Features used for collection of the time lag.

Feature name	Definition
Time	Time after nuclear division
Membrane intensity	Maximum intensity on the line connecting centers of the nuclei in membrane image
Membrane intensity ratio	The ratio of Membrane intensity to the previous time point
Membrane intensity thus far	Maximum Membrane intensity until this time point
Position of max intensity	The position of Max membrane intensity on the line connecting centers of the nuclei
Nuclear intensity	Mean intensity of the nuclei
Nuclear intensity ratio	The ratio of Nuclear intensity to the previous time point
Nuclear size	Total size (number of pixels) of the nuclei
Nuclear size ratio	The ratio of Nuclear size to the previous time point
Nuclear distance	Distance between the centers of nuclei
Nuclear distance ratio	The ratio of Nuclear distance to the previous time point

2 Supplementary videos

Supplementary Video 1: A segmentation result of cells that completed the cell cycle in an embryo.

Supplementary Video 2: The dynamics of ABplap and ABalpp.

The cellular regions of ABplap and ABalpp are colored in red. The video shows both cells do not contact.

Supplementary Video 3: The dynamics of ABplap and ABplpp.

The cellular regions of ABplap and ABplpp are colored in red. The video shows both cells do not contact.
